# Exploring the Competition between Halogen Bonding and CH Hydrogen Bonding in Bromoarenes Using an Aryldiyne Template

**DOI:** 10.1021/acsorginorgau.6c00009

**Published:** 2026-03-16

**Authors:** Maggie A. Schultz, Elijah T. Randazzo, Rachel A. Stindt, Shannon C. Riha, Joseph D. Scanlon, Eric Bosch, Nathan P. Bowling

**Affiliations:** † Department of Chemistry and Biochemistry, 14756 University of Wisconsin-Stevens Point, 2101 Fourth Ave., Stevens Point, Wisconsin 54481, United States; ‡ Department of Chemistry, 2719 Wabash College, 301 W Wabash Ave. Crawfordsville, Indiana 47933, United States; § Department of Chemistry and Biochemistry, 7471 Missouri State University, 901 S National Ave. Springfield, Missouri 65897, United States

**Keywords:** halogen bonding, CH hydrogen bonding, arylene ethynylene, inductive effects, electrostatic potential, DFT calculations

## Abstract

Hydrogens connected to benzene rings functionalized with electron-withdrawing groups are found to provide attractions with electron donors that are competitive with the halogen bond attraction to bromines on the same rings. An aryldiyne bridge that adequately templates this CH hydrogen bonding, along with the competing halogen bonding, provides an experimental pathway for looking at these interactions in a systematic way. Calculations using the M06–2*x*/6–311+G­(2d,p) density functional and basis set, along with mapping of the molecular electrostatic potentials using B3LYP/6–311++G**, support the experimental conclusion that CH hydrogen bonding to bromoarenes can be preferred to halogen bonding due to the greater positive potential on the surface of the hydrogen compared to that of the bromine. Computational evidence suggests a preference for the CH hydrogen bond conformer of the templated system regardless of the arrangement of electron-withdrawing (–F or −CF_3_) substituents on the haloarenes. At ∼5 kJ/mol or less, the energy differences between CH hydrogen bonding conformers and bromine-based halogen bonding conformers are often small, however, suggesting that selective crystal design using either interaction would be challenging.

## Graphical abstract

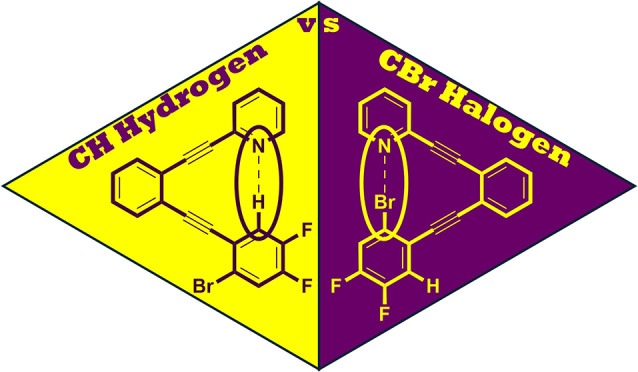

## Introduction

Though initial studies^1,2^ were met with some skepticism, hydrogen bonding from polarized C–H bonds has been accepted as a general phenomenon for decades.^3,4^ In recent years, polarized CH hydrogen bonding interactions have gained import in the chemistry community as they have proven useful in anion recognition^5,6^ and catalysis.^7,8^ Similarly, halogen bonding^9^ continues to emerge as a relevant driving force for crystal design,^10,11^ supramolecular assembly,^12–15^ and catalysis.^16–18^ Inductive effects from strong electron-withdrawing groups in the vicinity of a covalently bound bromine or iodine accentuate the electropositive region on the surface (σ hole) considerably, increasing the strength of the atoms’ attractions to electron donors (halogen bond acceptors).^9,19–22^ Hydrogens connected to electron-poor rings, such as fluoroarenes, experience a similar increase in electropositivity.^23^ Consequently, CH hydrogen bonding that results from increased electropositivity on a hydrogen atom can be a dominant supramolecular engineering force in its own right.^24–26^

The similar tendencies of halogens and hydrogens that are acted upon by nearby electron-withdrawing groups raise the question of what behavior (halogen bonding or CH hydrogen bonding) is likely to be observed when both are present on the same arene. In these cases, halogen bonding and CH hydrogen bonding can be used in concert for supramolecular assembly (Scheme 1A).^27,28^ When they do not act in concert, however, competition between C–X···N and C–H^·^···N interactions can lead to unanticipated outcomes.^29^

**Scheme 1:**
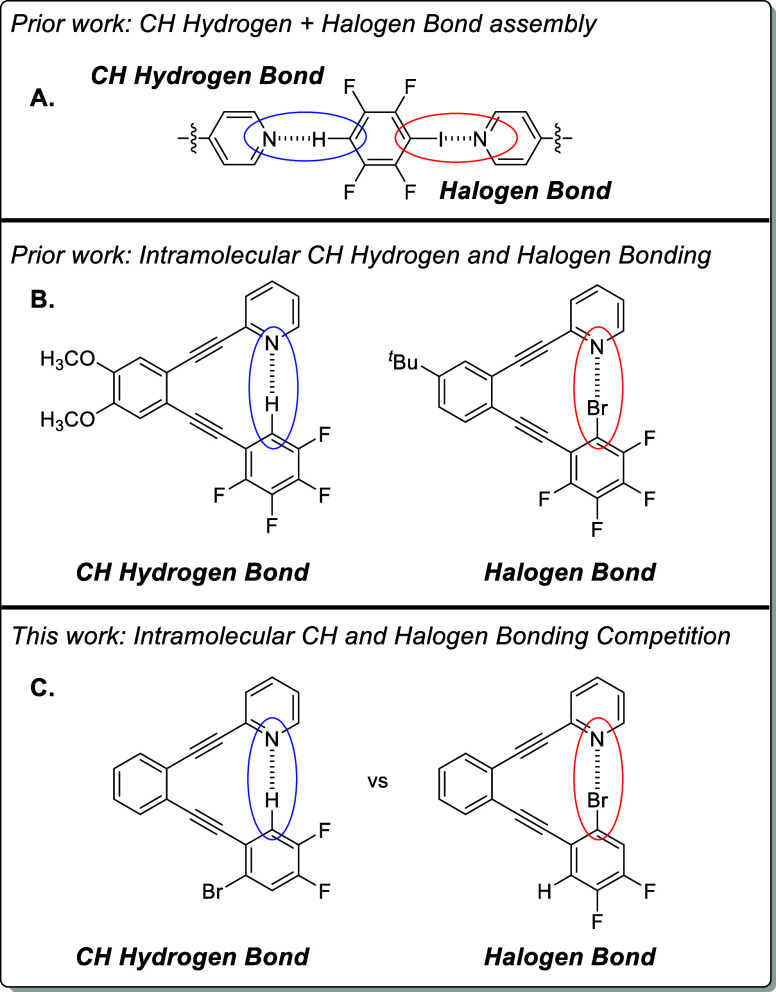
Prior and Current Work

As halogen bonding has risen to prominence as a predictable driving force for a variety of applications, there has been a general interest in learning about the relative favorability of halogen bond interactions compared to hydrogen bonding.^30^ While this has been extended to C–H···N interactions as well,^31^ there remains a need to tease out the relative favorability of C–X···N and C–H···N interactions when X and H are attached to the same arene. In prior studies, we have found an aryldiyne template to be beneficial for studying CH hydrogen^26^ and halogen bonds in the solid state^32^ and in solution (Scheme 1B).^33,34^ With a low energy barrier to rotation around the single bonds linking the alkynes and arenes,^35^ this kind of aryldiyne scaffold is well suited for studying competition between C–X···N and C–H···N interactions (Scheme 1C).

Four compounds (**1**–**4**) with the desired arrangement of halogens and hydrogens yielded crystals suitable for crystallography (Figure 1). In each, the X/H substituents have enhanced electropositivity due to the electron-withdrawing –F and −CF_3_ groups, respectively.

**Figure 1:**
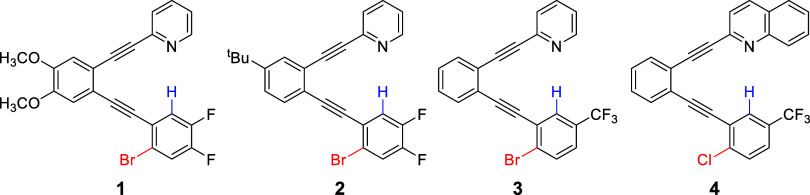
Intramolecular halogen/hydrogen bonding behavior of compounds **1–4** was characterized via X-ray crystallography and supported by computational chemistry.

## Experimental Section

### Synthesis and Solution Characterization Details

All solvents and reagents were used as received from commercial sources, unless otherwise specified. Routine ^1^H NMR (400 MHz), ^13^C­{^1^H} NMR (100 MHz), and ^19^F NMR (376 MHz) were performed on a Bruker Avance III 400 spectrometer using CDCl_3_ solvent. Flash chromatography was performed using SiliaFlash P60 or F60 silica gel. Thin-layer chromatography was performed using SiliaPlate 250 μm, F254 silica TLC plates. Each product was generated in a single step from terminal alkynes that we have previously developed (**10**–**13**) and commercially available haloarenes, as described. For reactions requiring heat, the reaction vessel rested on a metal block on a hot plate. Temperature was maintained by using a temperature probe linking the hot plate and the metal block. Mass spectrometry was performed using a Bruker amaZon Speed ion-trap mass spectrometer with positive electrospray ionization and auto MS­(n) mode.
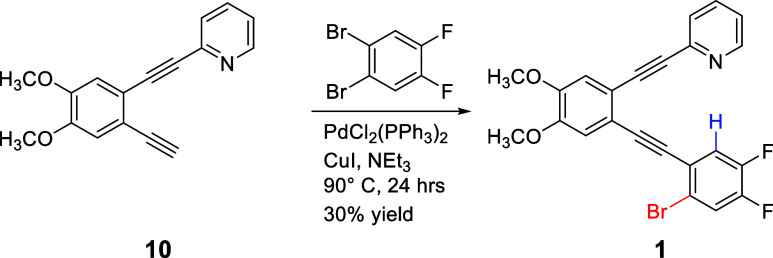


#### 2-((2-((2-Bromo-4,5-difluorophenyl)­ethynyl)-4,5-dimethoxyphenyl)­ethynyl)­pyridine (**1**)

Terminal alkyne **10** ^26^ (0.0318 g, 1.21 × 10^–4^ mol) was placed in a sealable storage vessel under argon. PdCl_2_(PPh_3_)_2_ (0.0042 g, 5.9 × 10^–6^ mol) and CuI (0.0020 g, 1.1 × 10^–5^ mol) were added, followed by deoxygenated NEt_3_ (15 mL). A 5× excess of commercially available 1,2-dibromo-4,5-difluorobenzene (0.1653 g, 6.08 × 10^–4^ mol) was added. The vessel was sealed under Ar and heated to 90 °C for 24 h. After being cooled to room temperature, the contents were rinsed into a separatory funnel using dichloromethane. The organic mixture was washed with an aqueous NH_4_Cl solution. After separation, the organic mixture was dried with anhydrous Na_2_SO_4_, filtered, and concentrated. The concentrated mixture was purified via flash chromatography (silica, 40% EtOAc/60% hexane, product *R*_f_ in 50:50 EtOAc/Hexane = 0.31) to yield the product as a light yellow crystalline solid (0.0163 g, 3.59 × 10^–5^ mol, 29.7% yield). ^1^H NMR (400 MHz, CDCl_3_, δ ppm): 8.65 (d, *J* = 4.8 Hz, 1 H), 7.72 (dd, *J* = 10.7, 8.1 Hz, 1 H), 7.68 (td, *J* = 7.7, 1.8 Hz, 1 H), 7.53 (d, *J* = 7.7 Hz, 1 H), 7.26 (m, 1 H), 7.13 (s, 1 H), 7.04 (s, 1 H), 3.95 (s, 3 H), 3.93 (s, 3 H). ^13^C­{^1^H} NMR (100 MHz, CDCl_3_, δ ppm): 150.1, 149.9 (dd, *J =* 256.1, 13.5 Hz), 149.62, 149.60, 149.3 (dd, *J =* 249.6, 13.0 Hz), 143.3, 136.1, 127.1, 122.8, 122.4 (dd, *J* = 7.9, 4.0 Hz), 122.0 (d, *J* = 19.6 Hz), 121.4 (d, *J =* 20.4 Hz), 119.5 (dd, *J* = 7.3, 3.5 Hz), 118.4, 118.2, 114.6, 114.0, 93.2 (d, *J* = 1.5 Hz), 91.7, 89.3, 87.9 (m), 56.1. ^19^F NMR (376 MHz, CDCl_3_, δ ppm): −133.2 (m), −138.4 (m). HRMS (ESI-Ion Trap) *m*/*z*: [M + H]^+^ Calcd for C_23_H_14_BrF_2_NO_2_H^+^ 454.0249; Found 454.0233.
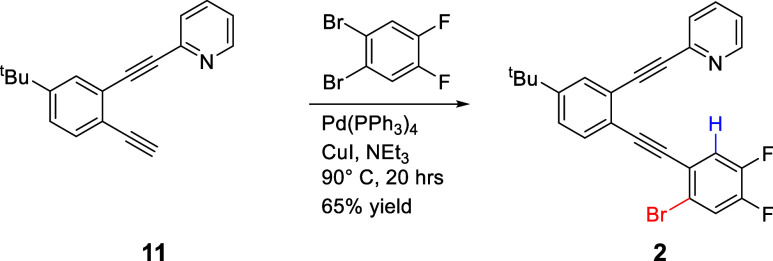


#### 2-((2-((2-Bromo-4,5-difluorophenyl)­ethynyl)-5-(*tert*-butyl)­phenyl)­ethynyl)­pyridine (**2**)

Terminal alkyne **11** ^34^ (0.0508 g, 1.96 × 10^–4^ mol) was placed in a sealable storage vessel under Ar. CuI (0.0018 g, 9.5 × 10^–6^ mol) and Pd­(PPh_3_)_4_ (0.0114 g, 9.87 × 10^–6^ mol) were added before the vessel was placed under vacuum (5 min) and then backfilled with Ar. A 5× excess of commercially available 1,2-dibromo-4,5-difluorobenzene (0.2604 g, 9.58 × 10^–4^ mol) was added along with deoxygenated NEt_3_ (10 mL). The vessel was sealed under Ar and heated at 90 °C for 20 h. After cooling to room temperature, the contents were rinsed into a separatory funnel using dichloromethane. The organic mixture was washed with an aqueous NH_4_Cl solution. After separation, the organic mixture was dried with anhydrous Na_2_SO_4_, filtered, and concentrated. The concentrated mixture was purified via consecutive flash chromatography columns (silica, 20% EtOAc/80% hexane then 5% EtOAc/95% hexane, *R*_f_ in 100% CH_2_Cl_2_ = 0.35) to yield the product as a yellow oil that later solidified (0.0572 g, 1.27 × 10^–4^ mol, 64.8% yield). ^1^H NMR (400 MHz, CDCl_3_, δ ppm): 8.66 (d, *J* = 4.9 Hz, 1 H), 7.70 (m, 3 H), 7.55 (m, 2 H), 7.41 (m, 2 H), 7.26 (m, 1 H), 1.34 (s, 9 H). ^13^C­{^1^H} NMR (100 MHz, CDCl_3_, δ ppm): 152.3, 150.1, 149.9 (dd, *J =* 256.0, 13.8 Hz), 149.3 (dd, *J =* 249.6, 13.0 Hz), 143.4, 136.1, 131.9, 129.7, 127.3, 126.2, 124.6, 122.9, 122.5 (dd, *J* = 8.0, 4.4 Hz), 122.4, 122.1 (d, *J* = 19.7 Hz), 121.4 (d, *J =* 20.3 Hz), 119.7 (dd, *J* = 7.0, 3.2 Hz), 93.1 (d, *J* = 2.1 Hz), 92.1, 89.8 (m), 88.2, 34.9, 31.0. ^19^F NMR (376 MHz, CDCl_3_, δ ppm): −133.1 (m), −138.4 (m). HRMS (ESI-Ion Trap) *m*/*z*: [M + H]^+^ Calcd for C_25_H_18_BrF_2_NH^+^ 450.0664; Found 450.0629.
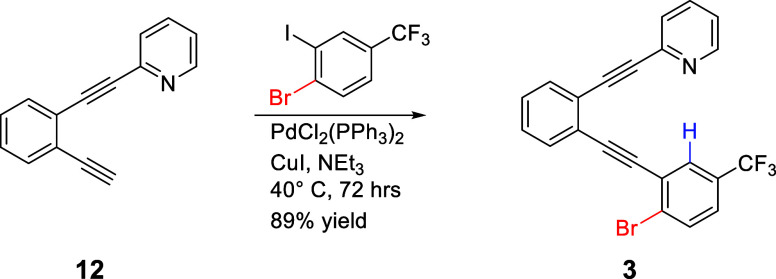


#### 2-((2-((2-Bromo-5-(trifluoromethyl)­phenyl)­ethynyl)­phenyl)­ethynyl)­pyridine (**3**)

Terminal alkyne **12** ^36^ (0.100 g, 4.92 × 10^–4^ mol) and commercially available 4-bromo-3-iodobenzotrifluoride (0.156 g, 4.45 × 10^–4^ mol) were placed with NEt_3_ (30 mL) in a sealable storage vessel equipped with a stir bar. The mixture was sparged with Ar for 20 min before PdCl_2_(PPh_3_)_2_ (0.0175 g, 2.49 × 10^–5^ mol) and CuI (0.0050 g, 2.6 × 10^–5^ mol) were added. The mixture was sealed under Ar and heated at 40 °C for 72 h. After it was cooled to room temperature, the mixture was rinsed into a separatory funnel using dichloromethane. After washing with an aqueous NH_4_Cl solution, the organic mixture was dried with anhydrous Na_2_SO_4_, filtered, and concentrated. The mixture was purified via flash chromatography (silica, 100% CH_2_Cl_2_, product *R*_f_ in 100% CH_2_Cl_2_ = 0.22) to reveal the product as a light yellow crystalline solid (0.170 g, 3.99 × 10^–4^ mol, 88.6% yield). ^1^H NMR (400 MHz, CDCl_3_, δ ppm): 8.65 (d, *J* = 4.9 Hz, 1 H), 8.00 (d, *J* = 2.2 Hz, 1 H), 7.72 (d, *J* = 8.4 Hz, 1 H), 7.65 (m, 3 H), 7.55 (d, *J* = 7.8 Hz, 1 H), 7.38 (m, 3 H), 7.25 (ddd, *J* = 7.6, 4.9, 1.2 Hz, 1 H). ^13^C­{^1^H} NMR (100 MHz, CDCl_3_, δ ppm): 150.2, 143.3, 136.1, 133.0, 132.4, 132.2, 130.6 (q, *J* = 3.8 Hz), 129.8 (q, *J* = 33.1 Hz), 129.3 (q, *J* = 1.7 Hz), 128.9, 128.8, 127.3, 126.5, 125.3, 125.2, 123.5 (q, *J* = 272.5 Hz), 123.0, 94.0, 93.0, 90.9, 87.5. ^19^F NMR (376 MHz, CDCl_3_, δ ppm): −62.8 ppm. HRMS (ESI-Ion Trap) *m*/*z*: [M + H]^+^ Calcd for C_22_H_11_BrF_3_NH^+^ 426.0100; Found 426.0085.
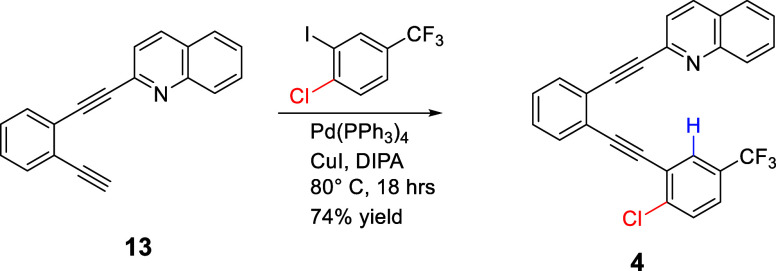


#### 2-((2-((2-Chloro-5-(trifluoromethyl)­phenyl)­ethynyl)­phenyl)­ethynyl)­quinoline (**4**)

A sealable storage vessel equipped with a stir bar was filled with Ar. Terminal alkyne **13** ^32^ (0.0748 g, 2.95 × 10^–4^ mol), Pd­(PPh_3_)_4_ (0.0165 g, 1.43 × 10^–5^ mol), and CuI (0.0053 g, 2.78 × 10^–5^ mol) were added, followed by diisopropylamine (10 mL) and tetrahydrofuran (10 mL). This mixture was sparged with Ar for 10 min. Commercially available 4-chloro-3-iodobenzotrifluoride (0.05 mL, 0.0919 g, 3.0 × 10^–4^ mol) was added via syringe. The vessel was sealed under Ar and heated to 80 °C for 18 h. After being cooled to room temperature, the mixture was transferred to a separatory funnel using aqueous NH_4_Cl solution and CHCl_3_. The organic mixture was dried with anhydrous Na_2_SO_4_, filtered, and concentrated. The mixture was purified via flash chromatography (silica, 100% CHCl_3_, product *R*_f_ in 100% CH_2_Cl_2_ = 0.60) to reveal the product as a light yellow solid (0.0937 g, 2.17 × 10^–4^ mol, 73.77% yield). ^1^H NMR (400 MHz, CDCl_3_, δ ppm): 8.13 (d, *J* = 8.4 Hz, 1 H), 8.12 (d, *J* = 8.4 Hz, 1 H), 7.92 (d, *J* = 2.1 Hz, 1 H), 7.80 (d, *J* = 8.2 Hz, 1 H), 7.74 (m, 2 H), 7.65 (dd, *J* = 6.0, 3.5 Hz, 1 H), 7.63 (d, *J* = 8.4 Hz, 1 H), 7.52 (m, 3 H), 7.40 (m, 2 H). ^13^C­{^1^H} NMR (100 MHz, CDCl_3_, δ ppm): 148.3, 143.4, 139.6, 136.1, 132.7, 132.4, 130.4 (q, *J* = 3.8 Hz), 130.1, 129.9, 129.2 (q, *J* = 33.2 Hz), 128.95, 128.94, 127.5, 127.25, 127.22, 125.9 (q, *J* = 3.6 Hz), 125.4, 125.0, 124.4, 124.1, 123.3 (q, *J* = 272.3 Hz), 94.6, 93.5, 89.1, 88.1. ^19^F NMR (376 MHz, CDCl_3_, δ ppm): −62.7. HRMS (ESI-Ion Trap) *m*/*z*: [M + H]^+^ Calcd for C_26_H_13_ClF_3_NH^+^ 432.0762; Found 432.0780.

### X-ray Crystallography Details

#### Crystallization

Each of the compounds **1**–**4** was recrystallized from absolute ethanol. Typically, 5–10 mg of **1** was placed in a screw cap glass vial, and 4 mL of absolute ethanol was added. The vial was capped and heated for a few minutes to ensure the solid totally dissolved. The vial was then allowed to cool to room temperature and left undisturbed for several days, during which time crystals suitable for XRD analysis formed.

#### XRD Analysis

For each compound, a single crystal was mounted on a Kryoloop using viscous hydrocarbon oil. Data were collected using a Bruker Apex1 CCD diffractometer equipped with Mo Kα radiation with λ = 0.71073 Å using APEX2 software.^37^ Data collection at 100 K was facilitated by a Kryoflex system with an accuracy of ±1 K. Initial data processing was carried out using SAINT with multiscan absorption correction performed with SADABS-2014.^37^ Structures were solved using SHELXT 2018/2 ^38^ and refined using SHELXL-2019/3 ^39^ with the program X-Seed as a graphical interface.^40^ All hydrogen atoms were located in the difference maps and were placed in idealized positions and refined with a riding model. Crystallographic details are collected in Table S1.

### Computational Details

#### Molecular Electrostatic Potential

The molecules 1-bromo-2-(3,4-dimethoxy-phenylethynyl)-4,5-difluorobenzene, 1-bromo-2-(4-*tert*-butyl-phenylethynyl)-4,5-difluorobenzene, 1-bromo-2-phenylethynyl-4-trifluoromethyl-benzene, and 1-chloro-2-phenylethynyl-4-trifluoromethyl-benzene were generated in the molecular modeling program Spartan’20 ^41^ in order to best evaluate the electrostatic potential on the portion of the molecule in each of **1**–**4** that serves as a hydrogen- or halogen-bond donor in this study. The molecules were geometry optimized using density functional theory (DFT) at the B3LYP/6–311++G** level subject to the constraint that the benzene rings were essentially coplanar to best mimic the conformation observed in cocrystals. The corresponding molecular electrostatic potential energy surfaces were calculated with an isovalue of 0.2 e/au^3^ and potentials are given in kJ/mol.

#### Computational Methods Using M062x Density Functional

All structures were optimized with the M062x density functional and the 6–311+G­(2d,p) basis set using *Gaussian16*. All of these were confirmed to be minima by vibrational frequency calculations. Structures were visualized using the WebMO program.^42–45^

## Results and Discussion

Compound **1** adopts a conformation in which the polarized C–H rather than C–Br points toward the pyridine nitrogen, suggesting that templated C–H hydrogen bonding may be favored in the solid state (Figure 2A). The 2.62 Å distance between pyridine N and C–H hydrogen is slightly longer than that observed when the arene has four fluorine substituents (2.39 Å) and relatively similar to a system with three (2.56 Å), highlighting the role of induction.^26^ The 2.62 Å distance between pyridine N and C–H hydrogen is accommodated by the template via alkyne bending, which allows this distance to shrink or expand as necessary to accommodate optimal N···H or N···X attractions. The most pronounced adjustment by the template in **1** is in the alkyne linker between the central arene and the haloarene, with inward bending to 174.8° and 175.2° to facilitate C–H hydrogen bond formation (Figure 2A). This attraction helps **1** retain near coplanarity, with twists of 10.5° and 6.6° of the pyridine and haloarene arms, respectively, relative to the central benzene (Figure 2B). In the crystal, the molecules are layered such that each unit is antiparallel and offset from those in the neighboring layer. Short contacts between pyridine N and hydrogens of methoxy groups on neighboring layers help organize and likely contribute to the slight out-of-plane twisting of the pyridyl unit (Figure 2B). Two types of self-complementary interactions help to organize the molecules within each layer (Figure 2C). The first—which is commonly found in veratrole-containing crystals^46^—is the self-complementary O···H–C interaction of the methoxy groups. On the other end of the molecule, self-complementary F···H interactions^47,48^ help align the arenes in the same plane. Interestingly, the bromine substituent—with a notable region of electropositivity due to induction from the fluorine groups, is not involved in any short contacts within the crystal lattice.

**Figure 2:**
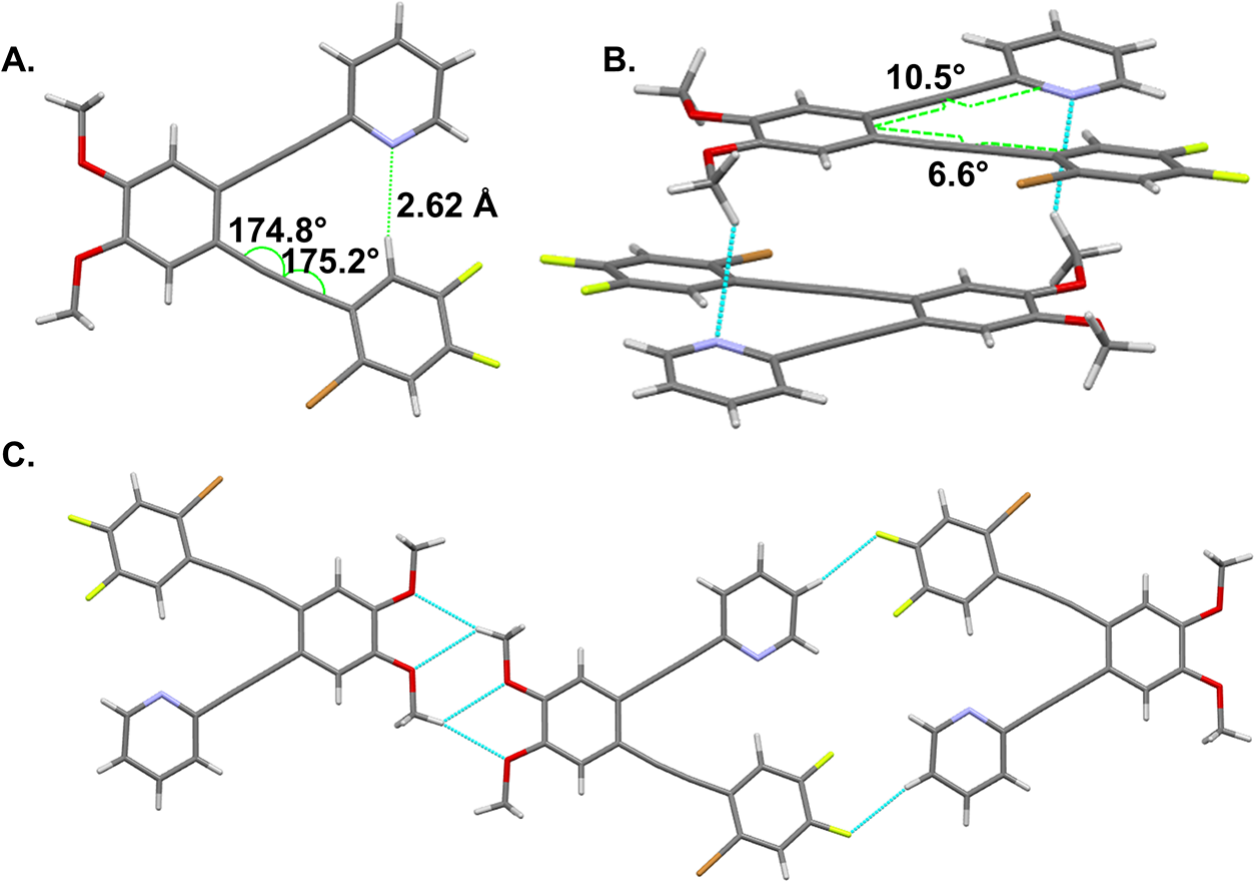
(A) Solid-state structure of **1**, along with (B) interlayer and (C) intralayer interactions.

The ostensible preference for C–H hydrogen bonding over C–Br halogen bonding that crystals of **1** convey is supported by electrostatic potential calculations of partial structures of **1** and **2** (differing only by the presence of methoxy or *tert*-butyl substituents on the remote ring). Using the B3LYP/6–311++G** level of theory and basis set, the surface of H shows greater electropositivity than the σ-hole of Br (Figure 3). Remote groups can alter the electrostatics of the interacting atoms, as exemplified by the slight decrease in electropositivity (122.1 vs 125.7 kJ/mol; 89.4 vs 91.5 kJ/mol) that results from conjugation with veratrole rather than the less donating *tert*-butylbenzene. These remote groups, however, do not impact the relative electropositivity difference between the bromine and hydrogen of interest.

**Figure 3:**
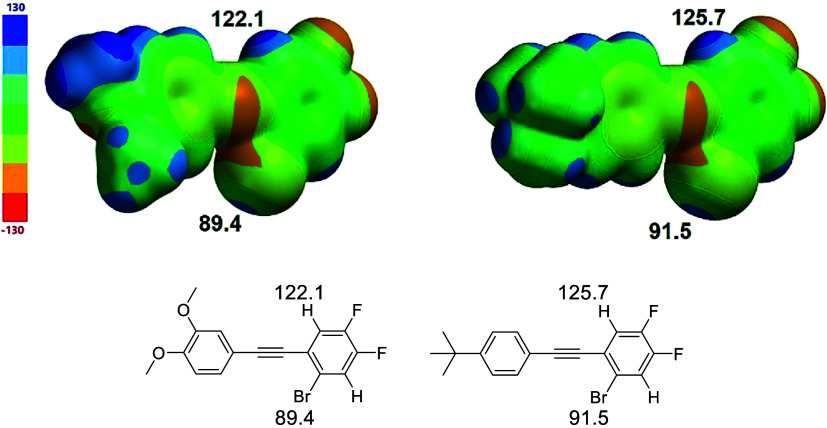
Molecular electrostatic potentials for partial structures of **1** and **2** show a greater concentration of electropositivity on H than on Br. Values in kJ/mol.

Further support for a preferential C–H hydrogen bonding conformation over C–Br halogen bonding comes from M06–2*x*/6–311+G­(2d,p) calculations in which the Gibbs energy for the C–H hydrogen bond conformer (0.00 kJ/mol) is lower than that of the halogen bond conformer (5.55 kJ/mol; Figure 4). The similar energies between the two conformers, **5-H** and **5-Br**, are consistent with the degree of attraction predicted, with the H···N attraction at 85.4% of the summed van der Waals radii (2.349 vs 2.75 Å^49^) and the Br···N interaction at 88.3% (3.003 vs 3.40 Å^49^).

**Figure 4:**
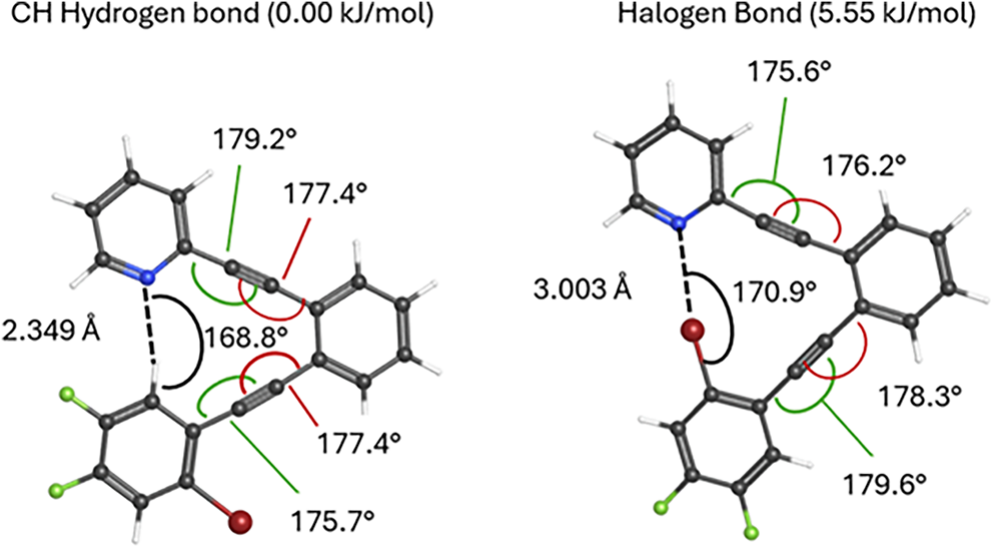
Gibbs energy optimization and vibrational frequency calculations were performed for **1** and **2**-type system **5** (lacking -OMe and -^*t*^Bu groups) with M06–2*x*/6–311+G­(2d,p).

The ability of the aryldiyne scaffold to template either intramolecular halogen or CH hydrogen bonding has been well established by solution,^24,33,34^ solid state,^26,32^ and computational studies.^32–34^ The current computed structures (Figure 4) nicely illustrate the utility of the aryldiyne scaffold for templating halogen and C–H hydrogen bond interactions, with slight bending of the alkynes away from linearity, providing a span suitable for either type of interaction. While the intent is that the scaffold is energetically innocent in the halogen bond vs CH hydrogen bond competition—a conclusion partially supported by the similar magnitude of distortions from linearity (175.7°, 177.4°, 177.4°, 179.2° vs 175.6°, 176.2°, 178.3°, 179.6°) for each—realistically, it is improbable that this scaffold provides zero bias. In addition to the small amounts of angle strain noted above, a small bias toward the CH hydrogen bond conformer could arise from nonoptimal angles for halogen bonding in the halogen bond conformer. Because they rely on electron interactions with a localized σ hole, strong halogen bonds require positioning of the acceptor (electron donor) near 180° relative to the C–X bond.^9^ While one might assess that the 170.9° N···Br–C angle predicted in Figure 4 is close enough to the idealized 180° bond angle necessary for strong halogen bonding, the small differences in energy between the halogen and CH hydrogen bonding conformations mandate some level of caution in dismissing bias that could be caused by the template.

Electrostatic calculations (Figure 3) infer that N···H–C hydrogen bonding is likely to be competitive with N···Br–C halogen bonding when the hydrogen and halogen are on the same arene. Though crystal packing, halogen bond alignment, and alkyne bending strain considerations all likely contribute to the overall stabilities, the preference for the CH hydrogen bond conformation in **1** is consistent with this prediction. Likewise, there are two unique structures in the asymmetric unit of compound **2**, each showing a preference for the C–H hydrogen bonding conformation over the halogen bonding conformation (Figure 5). The H···N distances (2.48 Å and 2.54 Å) are in line with those observed for **1**. As in **1**, these solid-state distances are slightly longer than those predicted for **5-H** in the gas phase (Figure 4, left). Similar to the case for **1** (Figure 2) and predictions from calculations (Figure 4), the most significant deviations from linearity arise in the alkynes linking the haloarene to the central benzene (Figure 5A). This inward bending is necessary to bring the hydrogen and nitrogen atoms into appropriate proximity for intramolecular C–H hydrogen bonding. Though the alkynes of the ethynylpyridine arm have greater linearity, the torsion of this arm is greater, with the pyridines out of plane from the central benzene by 11.8° and 16.7°, respectively (Figure 5B). Short contacts between the pyridine N and a CH hydrogen of a pyridine in the neighboring layer likely contribute to the 16.7° twist out of plane. In each of the two structures of the asymmetric unit, short contacts are consistent with some influence from π–π stacking between the pyridines of one layer and haloarenes of a neighboring layer. Though the Br of one unit has some short contacts, neither Br participates in halogen bonding, similar to compound **1**.

**Figure 5:**
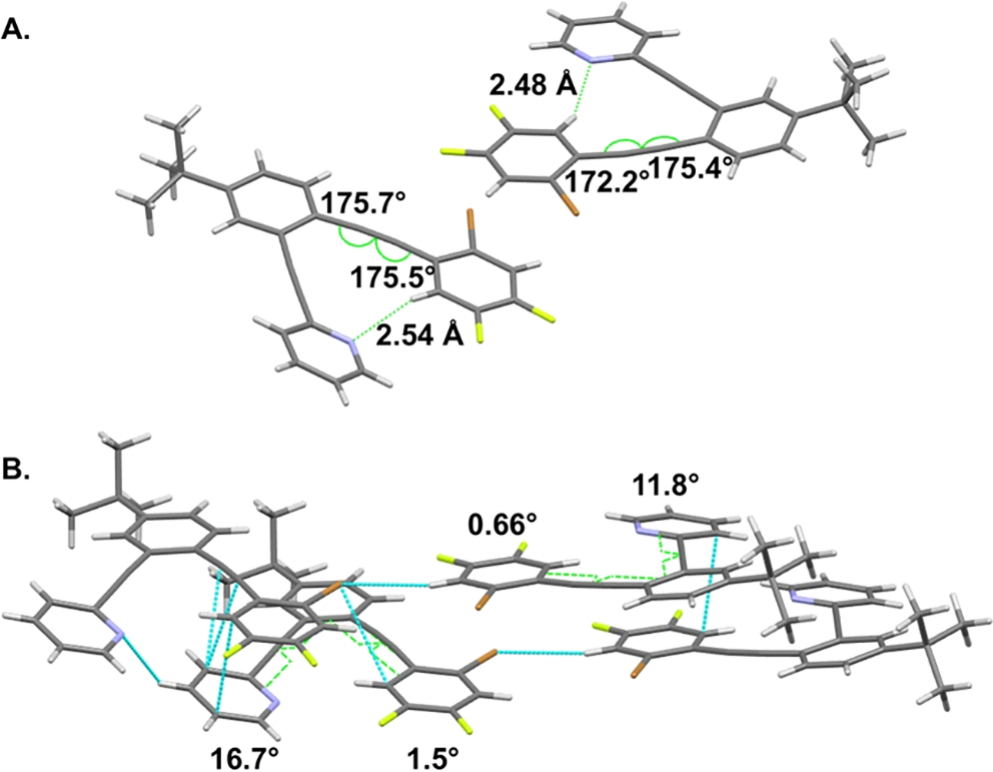
Analysis via X-ray crystallography reveals (A) two structures in the asymmetric unit, each displaying intramolecular CH hydrogen bonding. (B) Torsional angles reveal slight twisting out of the plane of the pyridine and haloarene arms.

Trifluoromethyl (−CF_3_) substituents have proven to be useful electron-withdrawing substituents for activating nearby halogen bond donors. Compound **3** was studied in order to determine if −CF_3_ groups have similar effects on neighboring hydrogens and how this CH hydrogen bond activation might compare to C–Br halogen bonding. To this end, compound **3** was crystallized from ethanol. Both the orientation of the C–H and the inward bend of the alkyne supporting the haloarene (176.3° and 175.1°) suggest some contributions from intramolecular CH hydrogen bonding (Figure 6A). The trifluoromethyl group complicates the evaluation of the CH hydrogen bonding strength as H···F interactions (2.54 Å) contribute to the overall stability. Optimization of both interactions while minimizing repulsion between N and F lone pairs results in H···N distance (2.84 Å) that is not necessarily indicative of a strong attraction. As with **1** and **2**, the pyridine arm shows a greater deviation from the plane of the benzene (12.0°) than the haloarene (3.5°)­(Figure 6B). Close contacts between molecules in the crystal lattice of **3** are not noteworthy, except that—as in **1** and **2**—halogen bonding does not play a significant role.

**Figure 6:**
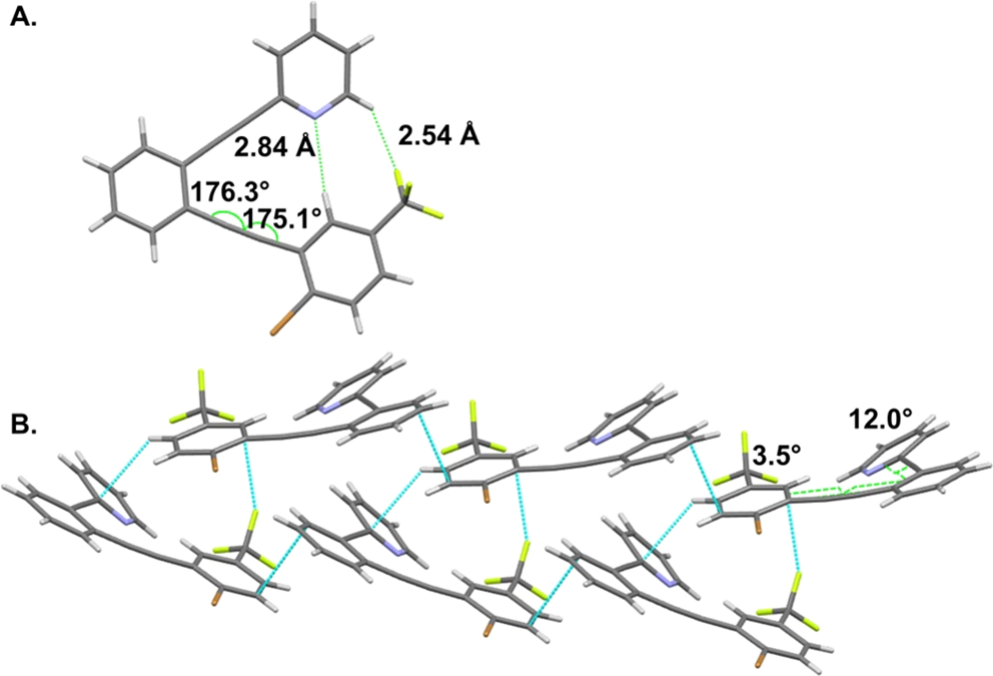
(A) Inward alkyne bending places the haloarene of **3** in position for H···N and H···F interactions. (B) Both the pyridine and the haloarene are twisted slightly out of the plane of the central benzene.

As with **1** and **2**, the apparent preference for the CH hydrogen bond conformation over the halogen bonding conformation in crystals of **3** is consistent with electrostatic potential calculations that show a greater electropositivity concentration on hydrogen (110.5 kJ/mol) than bromine (95.4 kJ/mol) (Figure 7). Interestingly, the hydrogen neighboring the bromine is more electropositive (122.1 kJ/mol) than the interacting hydrogen, raising the possibility that inter- rather than intramolecular CH hydrogen bonding might be more favorable. In the gas and solution phases, intramolecular interaction is likely favored due to the entropic cost of intermolecular association. Within the crystal, it is likely that the supporting H···F interaction plays a role in promoting the intramolecular attraction. The partial structure of chloro-derivative **4** demonstrates the known diminished σ-hole potential of chlorine (63.3 kJ/mol) compared with bromine (95.4 kJ/mol). As evidenced by the calculations, hydrogens on chlorinated rings, however, have CH hydrogen abilities comparable to those on brominated rings.

**Figure 7:**
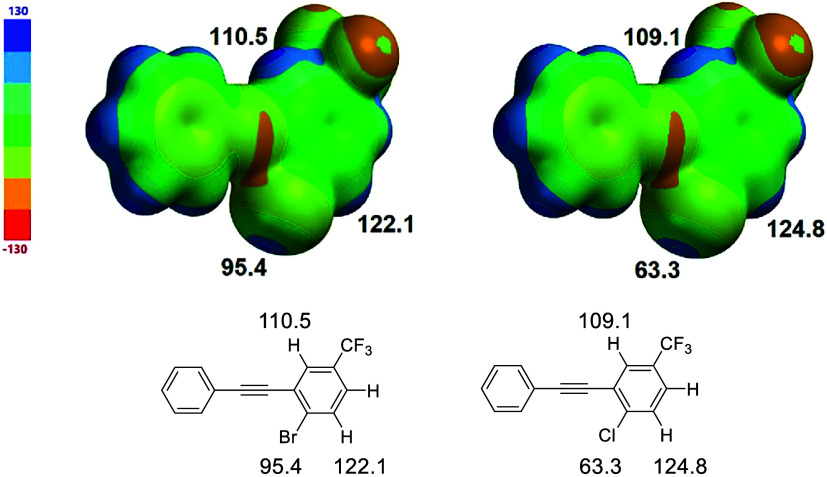
Molecular electrostatic potentials for partial structures of **3** and **4** emphasize the likelihood of competitive CH hydrogen bonding. Values in kJ/mol.

Calculations with the M06–2*x*/6–311+G­(2d,p) theory and basis set show the energetic and geometric differences between the intramolecular CH hydrogen bond conformation and the halogen bond conformers (Figure 8). Structures predicted for the trifluoromethyl-functionalized **3** are very similar to those of difluoro-functionalized **1** and **2**, in that the CH hydrogen bond conformer is predicted to be slightly more stable than the halogen bond conformer and that each is accessible through bending at the alkyne carbons. The predicted H···N distance (2.47 Å) in **3** is longer than that for **5** (2.35 Å), indicating a stronger inductive effect from the two fluorine substituents compared to a single trifluoromethyl, consistent with the calculated electrostatic potentials (Figures 3 and 7).

**Figure 8:**
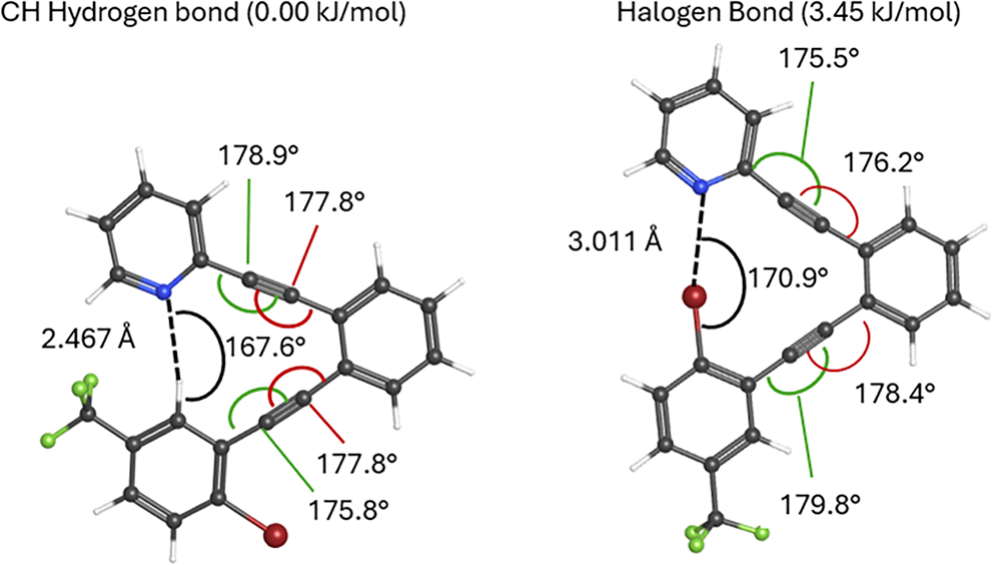
Gibbs energy optimization and vibrational frequency calculations were performed for system **3** with M06–2*x*/6–311+G­(2d,p).

Compound **4** pushes the limits of intramolecular interaction by (1) incorporating a chlorine, which is significantly less likely to form a halogen bond than bromine, and (2) adding extra steric bulk with quinoline rather than pyridine acting as the electron donor, increasing crowding with the trifluoromethyl substituent. Indeed, when **4** is crystallized from ethanol, no intramolecular interaction is observed (Figure 9). The quinoline N rotates outward from the central cavity, interacting with a haloarene H on a neighboring compound (H···N distance = 2.66 Å). Notable differences between **4**—with no intramolecular interaction—and compounds **1**-**3** include decreased coplanarity, with the quinoline arm twisted 23.0° out of plane from the central benzene, and a mixture of inward and outward bends at the alkyne carbons. While the magnitudes of the deviations from linearity are similar to those observed for **1**–**3**, the mix of inward bends (e.g., 172.6° for the alkyne carbon nearest the quinoline) and outward bends (176.4°) illustrates a distinct difference from those supporting intramolecular halogen or CH hydrogen bonding attractions.

**Figure 9:**
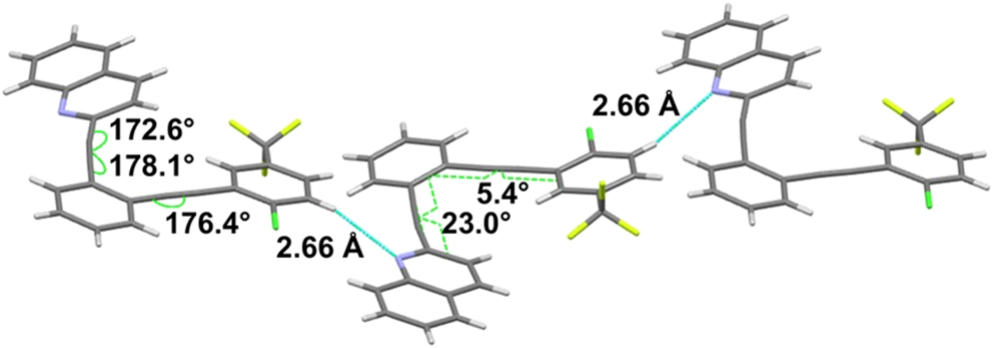
Characterization of **4** via X-ray reveals intermolecular, but no intramolecular, H···N interactions.

A general trend emerges from the experimental and computational results above, suggesting that hydrogens on the same benzene rings as bromine substituents have greater electropositivity, and therefore CH hydrogen bonding should be expected to be a competing factor any time that bromoarene halogen bonding is utilized (provided the arene has attached hydrogen atoms). In each of the bromoarene compounds (**1**–**3**) above, however, it must be noted that the hydrogens that are participating in intramolecular interactions with nitrogen are in closer proximity to the electron-withdrawing –F or −CF_3_ groups than the competing bromines. To investigate if this trend holds without this proximity effect, optimization and vibrational frequency calculations were performed with M06–2*x*/6–311+G­(2d,p) on compounds **6**–**9** and were compared to those on **5** and **3** (Table 1).

**Table 1:**
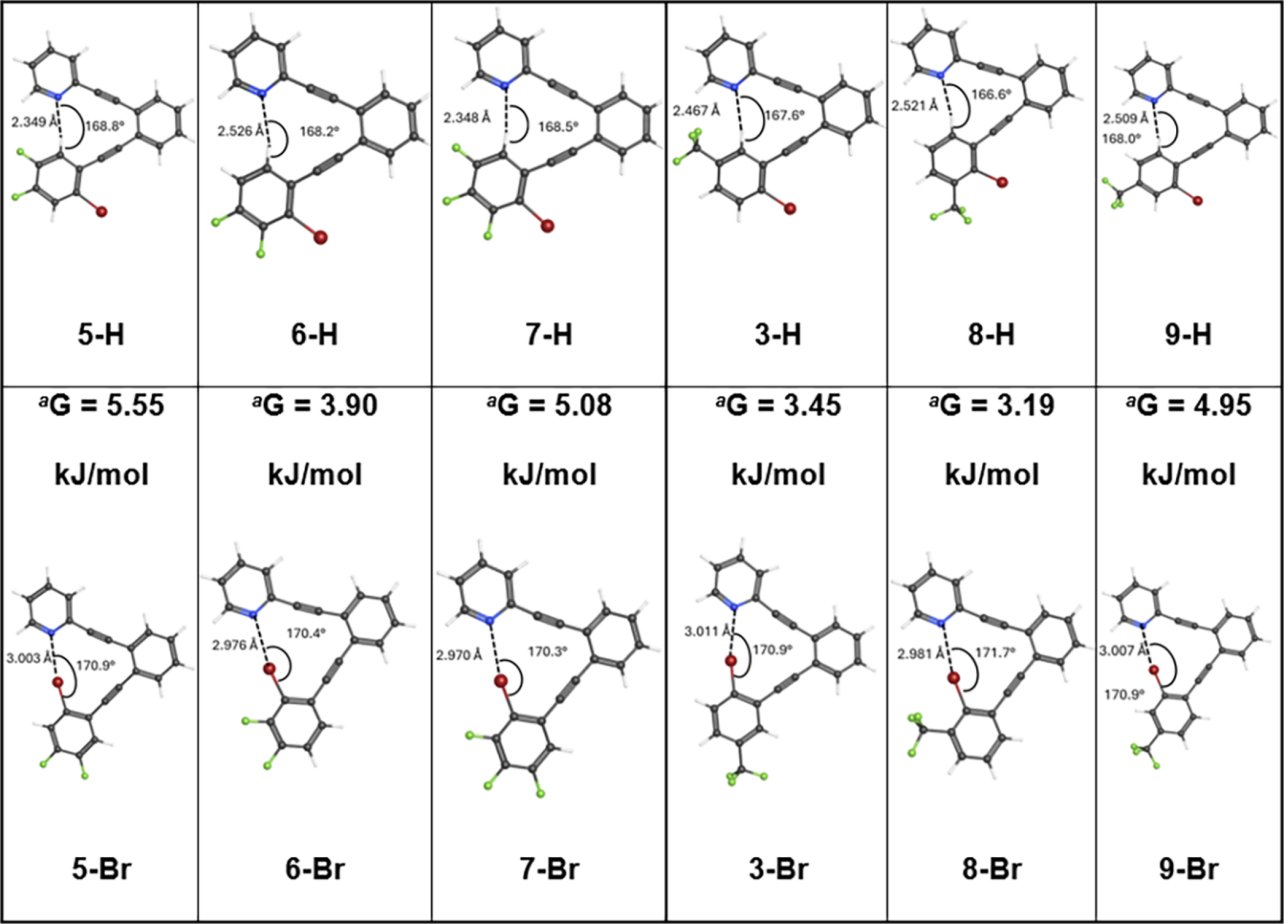
M062x/6-311+G­(2d,p) Structural Optimization and Energy Calculations for CH and Halogen Bonding Conformers

a Gibbs energies relative to the CH hydrogen bonding conformer at *G* = 0.00 kJ/mol.

In compounds **5**–**7**, there is a fluorine substituent in the *meta* position relative to both the Br and the H of interest in all structures (Table 1). Modifying **5** to **6**, the remaining fluorine is moved from *ortho* to the H to *ortho* to the Br. This results in a weakening of the CH hydrogen bond (**6-H** vs **5-H**) and a strengthening of the halogen bond (**6-Br** vs **5-Br**), as indicated by changes in interaction distances. While this shrinks the energy difference between the two to 3.90 kJ/mol, the CH hydrogen bond conformer is still predicted to be favored. In **7**, a fluorine is placed *ortho* to both Br and H of interest. Both **7-H** and **7-Br** have N···H/X distances that are very similar to **5-H** and **6-Br**, respectively, indicating that the installation of a fluorine substituent *para* to the group of interest has a much smaller impact than installation of a fluorine substituent in the *ortho* position. It is possible that the inductive electron-withdrawing effects of the remote fluorine are counterbalanced by π-electron donation from this substituent. As with **6-H**/**6-Br**, fluorine substituents *ortho* to the Br in **7** do not change the conformation preference.

Similarly, **8** and **9** represent the movement of the CF_3_ group from *ortho* to the H in **3** to *ortho* to the Br (**8**) and *meta* to both the H and the Br (**9**), respectively. Placing the CF_3_ group *ortho* to the Br in **8** indeed increases the halogen bond strength and decreases the CH hydrogen bond strength relative to **3**, indicated by the changes in the interaction distances. As with **5**–**7**, however, movement of the electron-withdrawing groups does not change the preference away from the CH hydrogen bond conformer. Like in **7**, the electron-withdrawing groups in **9** are equally distant from the Br and H of interest and therefore should not electrostatically bias the compound in favor of either conformation. In this case, as with **7**, the CH hydrogen bond conformer is predicted to be the preferred conformer.

## Conclusions

Interactions of lone pairs on an electron donor with hydrogen atoms on bromoarenes activated by electron-withdrawing groups can be as favorable as, or more favorable than, similar interactions with the bromine substituent. The use of a scaffold that has previously proven to be useful for templating both intramolecular CH hydrogen bonding and intramolecular halogen bonding provides a tool for putting these forces into direct competition. Though the template introduces variables that could potentially bias this competition due to crystal packing effects, bond angle strain concerns, and nonoptimal halogen bond angle consideration, the results in both the experimental and computational work are consistent with electrostatic potential calculations that predict CH hydrogen bonding is competitive with halogen bonding. Though the relative energies of the two types of interactions can be altered via placement of electron-withdrawing groups, the energies are similar, and the notable favorability of one attraction over another is improbable. Because this competition between halogen bonding and CH hydrogen bonding can cause complications that prevent controlled design, it is best to avoid hydrogen substituents on haloarenes under circumstances where halogen bonding is desired. Likewise, haloarenes should be avoided when CH hydrogen bonding is desired.

## Supplementary Material



## Data Availability

The data underlying this study are available in the published article and its Supporting Information.

## Notes

Table of Crystallographic Parameters, Cartesian Coordinates, and ^1^H, ^13^C, and ^19^F NMR spectra (PDF)
