# Supplementary material for: Exploring the Competition between Halogen Bonding and CH Hydrogen Bonding in Bromoarenes Using an Aryldiyne Template
Source: ACS Org Inorg Au. 2026 Mar 16;6(2):248–57. doi: 10.1021/acsorginorgau.6c00009 (PMC13047451; doi:10.1021/acsorginorgau.6c00009)

Supporting Information

Exploring the Competition Between Halogen Bonding and CH Hydrogen Bonding in Bromoarenes Using an Aryldiyne Template

Maggie A. Schultz[a], Elijah T. Randazzo[a], Rachel A. Stindt[a], Shannon C. Riha[a], Joseph D. Scanlon[b], Eric Bosch[c], Nathan P. Bowling[a]*

[a]Department of Chemistry and Biochemistry, University of Wisconsin-Stevens Point, 2101 Fourth Ave., Stevens Point, WI 54481, United States

[b]Department of Chemistry, Wabash College, 301 W Wabash Ave. Crawfordsville, IN 47933, United States

[c]Department of Chemistry and Biochemistry, Missouri State University, 901 S National Ave. Springfield, Missouri, 65897, United States

Table of Contents

Table of Crystallographic Data and Displacement Ellipsoids	S2-6
Cartesian Coordinates	S7-S18
^{1}H, ^{13}C, and ^{19}F NMR Spectra of **1-4**	S19-S26

Table S1. Table of Crystallographic Data

Structure number	Eb422, 1	Eb424, 2	Eb302, 3	Eb372, 4
**Crystal Data**				
formula	$C_{23}H_{14}BrF_2NO_2$	$C_{25}H_{18}BrF_2N$	$C_{22}H_{11}BrF_3N$	$C_{26}H_{13}ClF_3N$
FW (g/mol)	454.26	450.31	426.23	431.82
Crystal system, space group	Triclinic, *P*-1	Monoclinic, $P2_1/n$	Orthorhombic, *Pna*21	Orthorhombic, $P2_12_12_1$
a, b, c (Å)	8.9311 (1), 10.8395 (12), 11.5645 (12	21.0268 (18), 5.9123 (5), 33.159 (3)	9.7838(4), 9.6235(4), 18.9287(7)	4.1501(13), 21.344(7), 21.847(7)
a, b, g (deg)	109.947 (1), 106.271 (1), 100.731 (1)	90, 90.978 (2), 90	90, 90, 90	90, 90, 90
V (Å^3)	959.71 (18)	4121.7 (6)	1782.22 (12)	1935.2 (10)
Z	2	8	4	4
m (mm^{-1})	2.18	2.023	2.342	0.240
Crystal size (mm)	0.20 x 0.20 x 0.40	0.07 x 0.13 x 0.34	0.32 x 0.36 x 0.38	0.06 x 0.14 x 0.40
Density	1.572	1.451	1.589	1.482
**Data collection**				
T_{min}, T_{max}	0.5466, 0.7455	0.6289, 0.7455	0.6146, 0.7455	0.4424, 0.7455
No. of measured, independent and observed [**I** ≥ 2u(**I**)] reflections	12161, 4222, 3832	51722, 9217, 6379	21759, 3934, 3712	18031, 4406, 3332
R_{int}	0.0207	0.1068	0.0271	0.0994
**Refinement**				
$R[F^2 > 2\sigma(F^2)]$, $wR(F^2)$, S	0.0292, 0.855, 1.05	0.0533, 0.1203, 1.095	0.0224, 0.0553, 1.062	0.0669, 0.1626, 1.050
No. of reflections, parameters, restraints	4222, 268, 0	9217, 529, 0	3934, 245, 1	4406, 280, 0
$\Delta\rho_{max}$, $\Delta\rho_{min}$ (e Å^{-3})	0.96, -0.45	0.67, -0.58	0.51, -0.18	0.38, -0.51
CCDC deposition number	2476066	2476067	2476064	2476065

Figure S1. Displacement ellipsoid plot of **1** drawn at the 50% probability level.

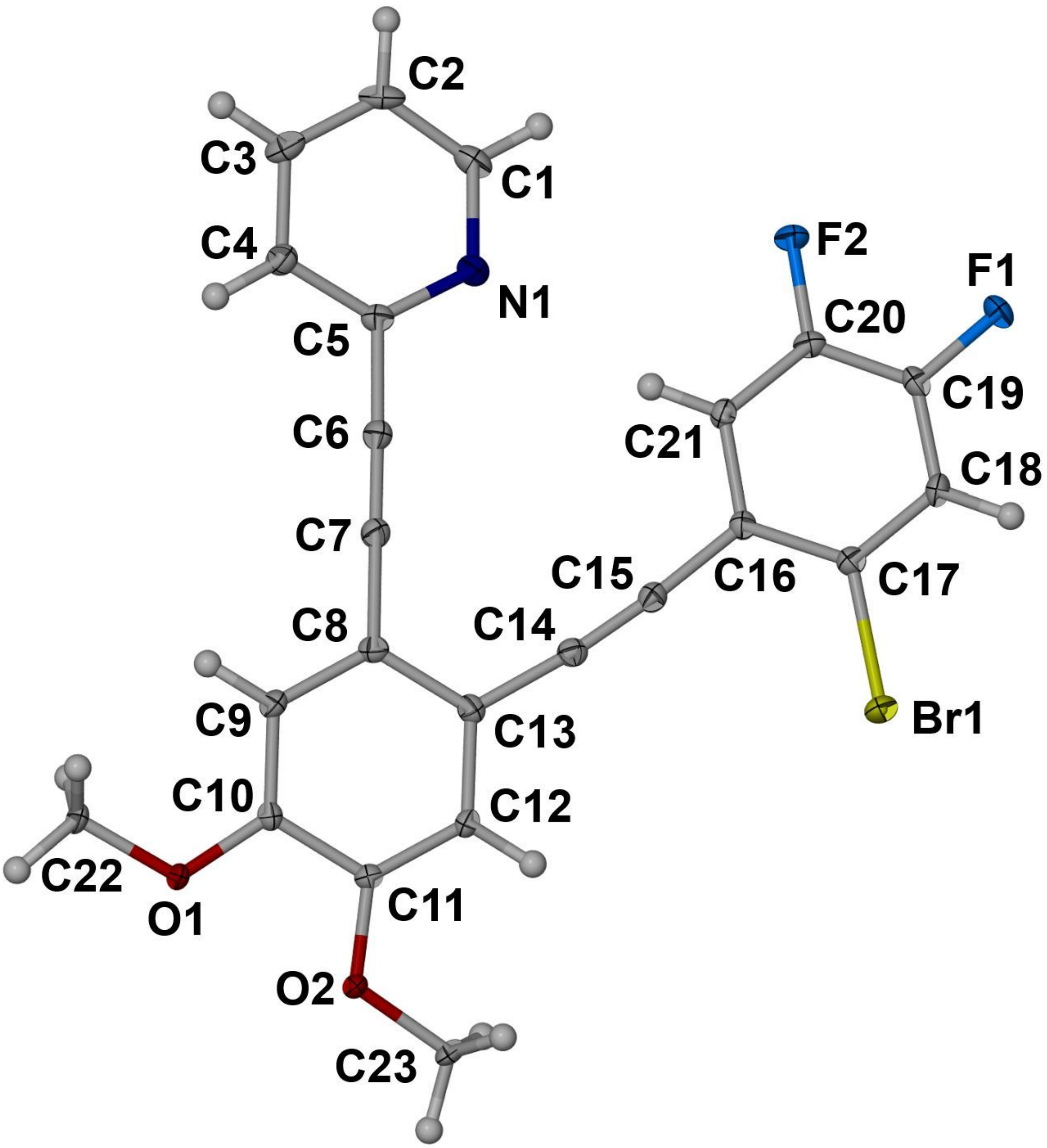

Figure S2. Displacement ellipsoid plot of **2** drawn at the 50% probability level.

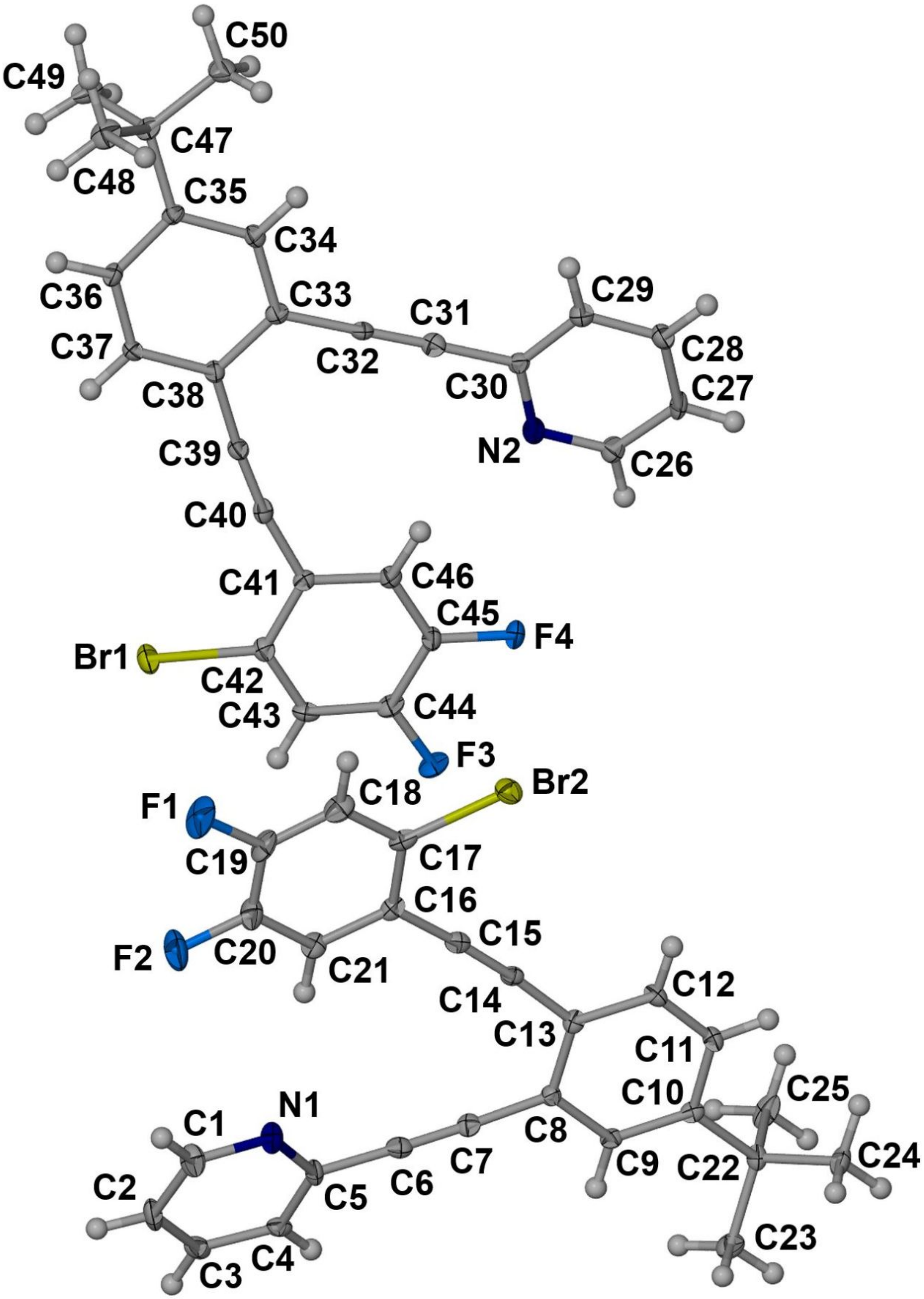

Figure S3. Displacement ellipsoid plot of **3** drawn at the 50% probability level.

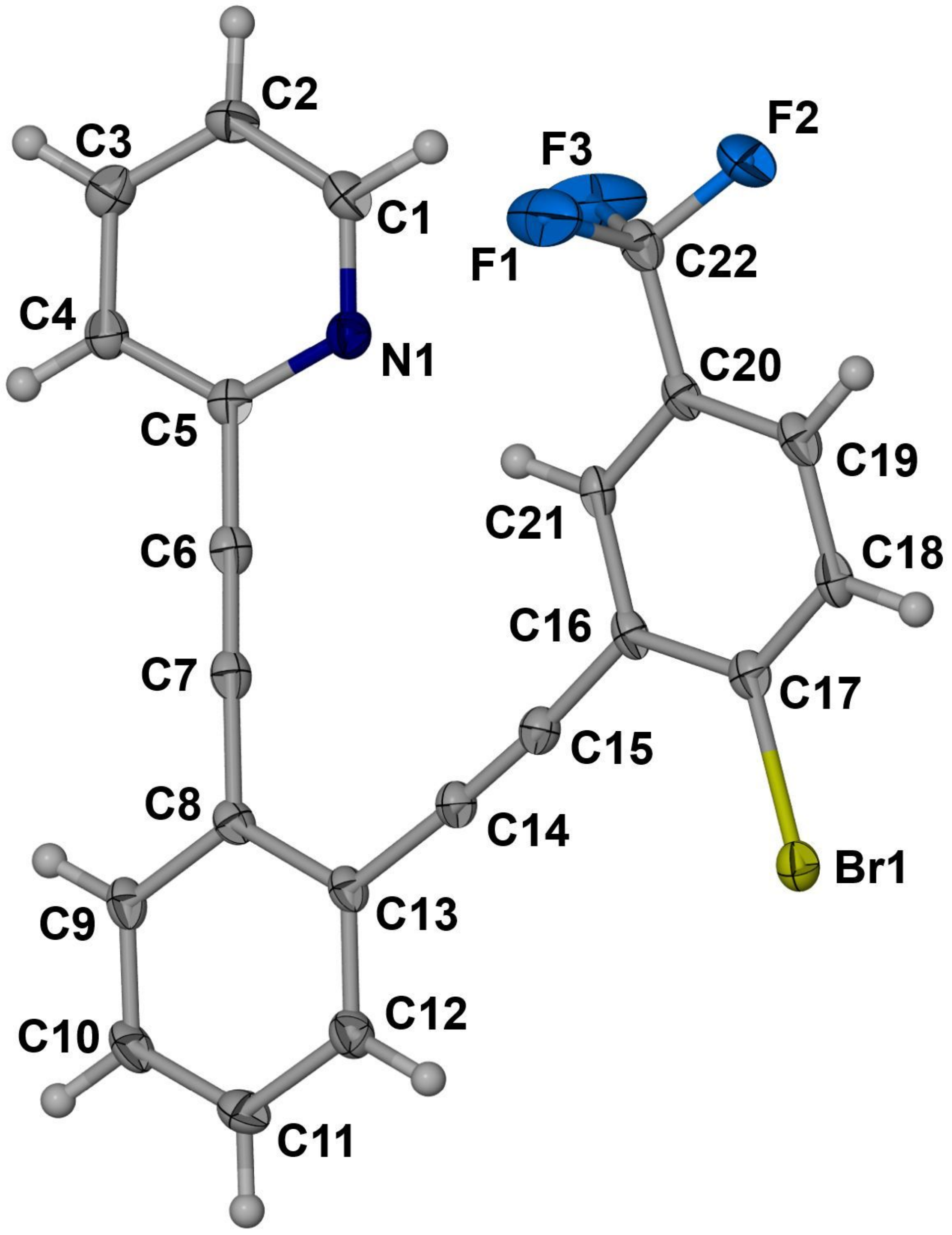

Figure S4. Displacement ellipsoid plot of **4** drawn at the 50% probability level.

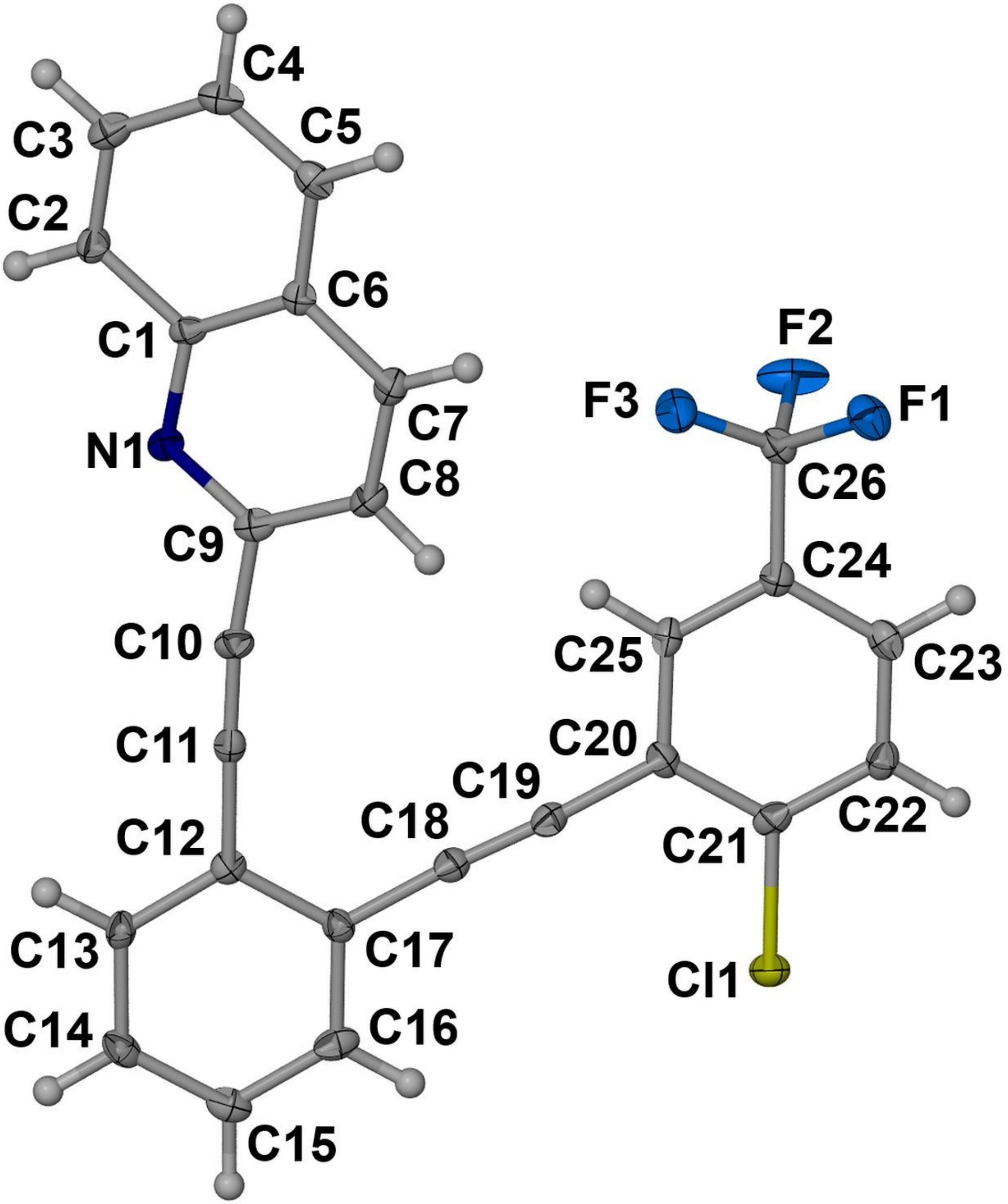

Table S2. Cartesian Coordinates

3-H

G = -3773.008933 hartrees

C	0.00000000	0.00000000	0.00000000
C	-1.47187100	-0.28576100	-0.00001500
C	-1.94422600	-1.58981400	-0.00007700
C	-3.31181600	-1.82111000	-0.00010900
C	-4.18935200	-0.75123000	-0.00008200
C	-3.72503500	0.57000300	-0.00002000
C	-4.59866800	1.69498300	0.00000500
C	-5.26506100	2.69519400	0.00000600
C	-5.99435400	3.91925800	-0.00000500
C	-7.39005600	3.92422400	0.00001500
C	-8.08716600	5.12053100	-0.00000100
C	-7.39907100	6.32943900	-0.00003600
C	-6.01434200	6.34128900	-0.00005400
C	-5.29547300	5.14479400	-0.00003900
C	-3.86834400	5.14963100	-0.00005600
C	-2.66642000	5.10762200	-0.00007600
C	-1.23126200	5.08435100	-0.00010100
C	-0.52000600	6.28588100	-0.00016600
C	0.86288100	6.23841300	-0.00017300
C	1.48771300	5.00155300	-0.00011500
C	0.69528100	3.85959900	-0.00005200
N	-0.63351000	3.88445300	-0.00004500
H	1.14929300	2.87570000	-0.00000100
H	2.56568800	4.91358800	-0.00011600
H	1.44230300	7.15308200	-0.00022100
H	-1.05724300	7.22433100	-0.00020600
H	-5.47084400	7.27693000	-0.00008100
H	-7.94405700	7.26451700	-0.00004800
H	-9.16943600	5.11173900	0.00001400
H	-7.91404400	2.97738900	0.00004100
C	-2.34448900	0.78938400	0.00001600
H	-1.96713300	1.80842800	0.00006900
Br	-6.04698500	-1.10103200	-0.00013100
H	-3.69782100	-2.83077600	-0.00015400
H	-1.25302600	-2.42142300	-0.00009800
F	0.36940600	0.71444900	1.07336500
F	0.36947500	0.71420900	-1.07350400
F	0.73378400	-1.11800000	0.00014200

3-Br

G = -3773.007619 hartrees

C	0.00000000	0.00000000	0.00000000
C	1.44886400	-0.38473400	0.00007500
C	1.77483000	-1.73735900	0.00020400

C	3.10294900	-2.12212500	0.00032200
C	4.10484900	-1.16066600	0.00031600
C	3.78759000	0.20276400	0.00019100
C	4.80245200	1.20458500	0.00019500
C	5.66148200	2.04650200	0.00020800
C	6.65134600	3.07338400	0.00022900
C	6.25081300	4.41274600	0.00026100
C	7.18624700	5.43201900	0.00028000
C	8.54380600	5.12932600	0.00026700
C	8.95720400	3.80909200	0.00023400
C	8.02623100	2.76585300	0.00021700
C	8.50180600	1.41883800	0.00018400
C	8.97632000	0.31327200	0.00015100
C	9.64567000	-0.95823200	0.00008700
C	11.0431000	-0.98959900	-0.00032200
C	11.6813250	-2.21694400	-0.00037700
C	10.9128590	-3.37095300	-0.00002500
C	9.52998700	-3.23920000	0.00036700
N	8.90081100	-2.06996300	0.00042900
H	8.89349400	-4.11794400	0.00064100
H	11.3670970	-4.35247800	-0.00005500
H	12.7627180	-2.27102000	-0.00069400
H	11.5975530	-0.06123100	-0.00059900
H	10.0107770	3.56247500	0.00022500
H	9.27919400	5.92339100	0.00028300
H	6.85785600	6.46327600	0.00030600
H	5.19224700	4.63679900	0.00027000
C	2.43847400	0.57714400	0.00007000
H	2.18363700	1.62796700	-0.00003300
Br	5.91363500	-1.69083700	0.00047600
H	3.37178700	-3.16945100	0.00041800
H	0.99063600	-2.48425700	0.00020400
F	-0.17563900	1.32432500	-0.00054900
F	-0.63927600	-0.48581700	1.07397400
F	-0.63941100	-0.48673700	-1.07347200

5-H

G = -3634.446458 hartrees

C	0.00000000	0.00000000	0.00000000
C	1.38224000	0.10948700	0.00039300
C	1.98858300	1.34772100	0.00082900
C	1.19471900	2.48356400	0.00086300
C	-0.19969800	2.39267200	0.00046300
C	-1.05087400	3.53514000	0.00046100

C	-1.83989900	4.44205800	0.00042800
C	-2.82399600	5.47296100	0.00037700
C	-2.45357100	6.81873600	0.00046500
C	-3.41782700	7.81240800	0.00040800
C	-4.76769200	7.47645400	0.00026300
C	-5.15200200	6.14623000	0.00017600
C	-4.19334700	5.13174000	0.00023000
C	-4.58120100	3.75834300	0.00014000
C	-4.85467100	2.58725100	0.00007200
C	-5.16133300	1.18556200	-0.00000900
C	-6.48796700	0.75115300	0.00017300
C	-6.73974100	-0.60984100	0.00008600
C	-5.66869200	-1.48995000	-0.00017400
C	-4.38266700	-0.96304200	-0.00034400
N	-4.12211300	0.33915400	-0.00027000
H	-3.51735200	-1.61796500	-0.00055500
H	-5.81809700	-2.56108500	-0.00024900
H	-7.75818000	-0.97740300	0.00021900
H	-7.28885800	1.47771000	0.00037300
H	-6.19943900	5.87468000	0.00006300
H	-5.52098600	8.25353600	0.00021800
H	-3.11728300	8.85216600	0.00047600
H	-1.40067700	7.06866100	0.00057700
C	-0.79202600	1.12189200	0.00002800
H	-1.87287000	1.01619100	-0.00026500
Br	2.04249200	4.17307700	0.00148100
H	3.06759400	1.41064400	0.00113300
F	2.12263300	-0.99812400	0.00034700
F	-0.54906000	-1.21779200	-0.00041400

5-Br

G = -3634.444345 hartrees

C	0.00000000	0.00000000	0.00000000
C	-0.12653600	-1.38065600	0.00014500
C	-1.37154000	-1.97252100	0.00034600
C	-2.50390300	-1.17227600	0.00040000
C	-2.39407400	0.22385600	0.00025400
C	-3.54784900	1.06153800	0.00030100
C	-4.52584800	1.76175500	0.00033000
C	-5.66075900	2.62555800	0.00035200
C	-5.46918200	4.01049500	0.00038400

C	-6.54876800	4.87562100	0.00039500
C	-7.84460400	4.37008300	0.00037200
C	-8.05195400	3.00230500	0.00033800
C	-6.97302100	2.11277800	0.00033100
C	-7.23840400	0.70910000	0.00029500
C	-7.53913600	-0.45584500	0.00027000
C	-8.00526400	-1.81506600	0.00020200
C	-9.38120900	-2.06152100	-0.00045300
C	-9.82248900	-3.37261900	-0.00051100
C	-8.88533600	-4.39439000	0.00008200
C	-7.53935100	-4.05109300	0.00071000
N	-7.09809400	-2.79883900	0.00077500
H	-6.77495900	-4.82115100	0.00118800
H	-9.18285700	-5.43419000	0.00006200
H	-10.8825810	-3.59293500	-0.00101600
H	-10.0721340	-1.22969800	-0.00090000
H	-9.05569900	2.59811100	0.00032100
H	-8.69233300	5.04289200	0.00038000
H	-6.38105000	5.94485400	0.00042200
H	-4.45712700	4.39328600	0.00040000
C	-1.11750400	0.79962900	0.00005400
H	-0.99994000	1.87470400	-0.00006200
Br	-4.21111500	-1.97258400	0.00067200
H	-1.44508900	-3.05118600	0.00045700
F	0.97422600	-2.13110600	0.00008800
F	1.22055200	0.53870500	-0.00019400

6-H

G = -3634.443711 hartrees

C	0.00000000	0.00000000	0.00000000
C	-1.37257500	0.13793400	-0.00017500
C	-1.96434700	1.39055500	-0.00033000
C	-1.17804700	2.52791500	-0.00031000
C	0.21732700	2.41011100	-0.00013400
C	1.06996400	3.55110700	-0.00010300
C	1.85509100	4.46131000	-0.00007300
C	2.83281400	5.49765400	-0.00009200
C	2.45442400	6.84132400	-0.00022400
C	3.41263800	7.84071800	-0.00023500
C	4.76458600	7.51297600	-0.00011400

C	5.15655100	6.18510600	0.00001800
C	4.20376500	5.16482600	0.00003100
C	4.60422700	3.79497400	0.00016800
C	4.90306100	2.63003200	0.00027300
C	5.28188100	1.24634100	0.00034300
C	6.63411000	0.89595600	0.00043400
C	6.97365600	-0.44509000	0.00050300
C	5.96024100	-1.39104800	0.00048100
C	4.64458700	-0.94498900	0.00039000
N	4.29702800	0.33706000	0.00032200
H	3.82659100	-1.65802100	0.00037100
H	6.17638300	-2.45074200	0.00053400
H	8.01353900	-0.74653500	0.00057400
H	7.38625900	1.67284200	0.00044800
H	6.20557500	5.91970500	0.00011300
H	5.51321600	8.29453800	-0.00012300
H	3.10581600	8.87864700	-0.00033800
H	1.40004900	7.08490000	-0.00031700
C	0.79286900	1.13449400	0.00002100
H	1.87355500	1.04810200	0.00015900
Br	-2.02782500	4.20408900	-0.00052700
F	-3.28997000	1.47189700	-0.00049600
F	-2.16459900	-0.93443900	-0.00019900
H	0.42762000	-0.99343100	0.00011800

6-Br

G = -3634.442224 hartrees

C	0.00000000	0.00000000	0.00000000
C	-0.16231100	-1.36984100	0.00016000
C	0.93251000	-2.21907500	0.00020900
C	2.21619000	-1.70602100	0.00009800
C	2.40203400	-0.31522100	-0.00006900
C	3.71468800	0.24242900	-0.00020300
C	4.82428100	0.70685100	-0.00029100
C	6.12393900	1.29433200	-0.00004600
C	6.24823100	2.68695100	0.00001200
C	7.49432400	3.28792400	0.00022400
C	8.64378100	2.50475700	0.00038000
C	8.53894100	1.12529000	0.00032000
C	7.28796500	0.50047900	0.00010700
C	7.23129200	-0.92686500	0.00003500
C	7.26131700	-2.12960400	-0.00001400

C	7.40293100	-3.55944400	0.00015000
C	8.68464900	-4.11674400	0.00034300
C	8.81112900	-5.49442700	0.00048000
C	7.66348700	-6.27237100	0.00041900
C	6.43283500	-5.62801000	0.00021900
N	6.29333800	-4.30758200	0.00008800
H	5.51099700	-6.20022700	0.00016300
H	7.71299500	-7.35276100	0.00052000
H	9.79177000	-5.95344000	0.00063100
H	9.54903800	-3.46703600	0.00038000
H	9.42636500	0.50613500	0.00043400
H	9.62085900	2.97019500	0.00054600
H	7.57062500	4.36756100	0.00026700
H	5.34783500	3.28699700	-0.00011400
C	1.28227500	0.52125900	-0.00011400
H	1.43361000	1.59179400	-0.00024400
Br	3.68590700	-2.87306500	0.00019800
F	0.71954300	-3.53036700	0.00036800
F	-1.38206500	-1.90770200	0.00027200
H	-0.87605600	0.63389100	-0.00003300

7-H

G = -3733.688679 hartrees

C	0.00000000	0.00000000	0.00000000
C	-1.36813200	0.21339500	-0.00020600
C	-1.85008900	1.51094200	-0.00042500
C	-0.98138900	2.58879500	-0.00044100
C	0.39936800	2.36335300	-0.00023600
C	1.34079200	3.43240800	-0.00023700
C	2.20278600	4.27004500	-0.00022600
C	3.27248400	5.21175000	-0.00017500
C	3.02044300	6.58452700	-0.00025100
C	4.06784700	7.49014900	-0.00018800
C	5.38300000	7.03719700	-0.00005000
C	5.65002800	5.67847400	0.00002500
C	4.60648700	4.75153500	-0.00003700
C	4.87298800	3.34949500	0.00002600
C	5.04540100	2.15932800	0.00006800
C	5.23596100	0.73713700	0.00016800
C	6.52313700	0.19692500	0.00039900
C	6.66409600	-1.17996300	0.00048000

C 5.52526400 -1.97040500 0.00032800
C 4.28613200 -1.34123000 0.00009800
N 4.13149900 -0.02218800 0.00001700
H 3.37099900 -1.92443100 -0.00003000
H 5.58741600 -3.05011900 0.00038300
H 7.64947600 -1.62863800 0.00065900
H 7.38008600 0.85641300 0.00051000
H 6.66977400 5.31661100 0.00012900
H 6.20142600 7.74536300 -0.00000100
H 3.85938200 8.55216900 -0.00024600
H 1.99343400 6.92547500 -0.00035600
C 0.88586200 1.05101600 -0.00001100
H 1.95363100 0.85508300 0.00016200
Br -1.70288800 4.32283300 -0.00074000
F -3.16460000 1.68859500 -0.00061600
F -2.21452400 -0.80763500 -0.00019400
F 0.44001500 -1.25941200 0.00021300

7-Br
G = -3733.686746 hartrees
C 0.00000000 0.00000000 0.00000000
C -0.01131500 -1.38428200 0.00004600
C 1.19059700 -2.07131000 0.00012700
C 2.39897500 -1.39700900 0.00016200
C 2.40039400 0.00450300 0.00011300
C 3.62800800 0.72952900 0.00014600
C 4.66788200 1.33369000 0.00016800
C 5.87946000 2.08635100 0.00013600
C 5.81907800 3.48300800 0.00004200
C 6.97563300 4.24211300 0.00001300
C 8.21754900 3.61596400 0.00008000
C 8.29503700 2.23458900 0.00017300
C 7.13697300 1.45109300 0.00020100
C 7.26675600 0.02856100 0.00029900
C 7.45243000 -1.16011500 0.00037000
C 7.77798300 -2.55971000 0.00032500
C 9.12084600 -2.94626300 0.00028400
C 9.42458800 -4.29605200 0.00025400
C 8.38731300 -5.21591100 0.00026800
C 7.08353000 -4.73633200 0.00031200
N 6.77439800 -3.44504000 0.00034100

H	6.24349600	-5.42303100	0.00032600
H	8.57623600	-6.28078400	0.00024600
H	10.4563600	-4.62427800	0.00022200
H	9.89393400	-2.19021100	0.00027600
H	9.25620200	1.73763600	0.00022600
H	9.12507400	4.20563500	0.00005900
H	6.90993300	5.32239500	-0.00006100
H	4.84766700	3.95958300	-0.00000800
C	1.18574200	0.69709400	0.00003200
H	1.16747400	1.77782800	-0.00000600
Br	4.00784300	-2.36387200	0.00027900
F	1.14670200	-3.39727700	0.00017100
F	-1.15833400	-2.04988100	0.00001400
F	-1.16697800	0.64312300	-0.00007700

8-H

G = -3773.002477 hartrees

C	0.00000000	0.00000000	0.00000000
C	-1.50059900	-0.14837800	0.00003800
C	-2.34252100	0.96141800	0.00001700
C	-3.73104400	0.79052100	0.00005300
C	-4.63881200	1.88928500	0.00003700
C	-5.47819900	2.74961700	0.00002700
C	-6.50699900	3.73546500	0.00001700
C	-6.19271700	5.09556700	0.00000700
C	-7.19731000	6.04825700	-0.00000300
C	-8.53190800	5.65608500	-0.00000400
C	-8.86047800	4.31096300	0.00000500
C	-7.86019000	3.33718600	0.00001600
C	-8.19417700	1.94952900	0.00002400
C	-8.43606100	0.77144100	0.00003100
C	-8.74229200	-0.63020900	0.00003400
C	-10.0737740	-1.05256600	-0.00001500
C	-10.3407200	-2.41001900	-0.00001200
C	-9.27799600	-3.30026900	0.00003900
C	-7.98814300	-2.78417700	0.00008600
N	-7.71020100	-1.48540200	0.00008400
H	-7.13299600	-3.45219500	0.00012600
H	-9.43687700	-4.37005600	0.00004300
H	-11.3628530	-2.76705600	-0.00005000
H	-10.8667470	-0.31739900	-0.00005500

H	-9.89575900	3.99624300	0.00000400
H	-9.31718000	6.40084700	-0.00001200
H	-6.94030400	7.09961300	-0.00001100
H	-5.15095100	5.38842900	0.00000700
C	-4.26104700	-0.50658800	0.00010700
C	-3.42167500	-1.60305600	0.00012700
C	-2.04558600	-1.42553600	0.00009400
H	-1.38309500	-2.27937600	0.00011000
H	-3.83658400	-2.60217600	0.00016900
H	-5.33891300	-0.62832100	0.00012900
Br	-1.64944100	2.71952200	-0.00006400
F	0.60647200	-1.19420200	0.00002400
F	0.44253100	0.65522400	-1.07759300
F	0.44259100	0.65529300	1.07749800

8-Br

G = -3773.001261 hartrees

C	0.00000000	0.00000000	0.00000000
C	-0.75991100	-1.30288800	-0.00000500
C	-2.15350900	-1.32432500	-0.00003100
C	-2.82825300	-2.55344300	-0.00003500
C	-4.25369700	-2.62039600	-0.00006200
C	-5.45389200	-2.69997900	-0.00008500
C	-6.87417200	-2.83220100	-0.00011000
C	-7.44457900	-4.10857000	-0.00009300
C	-8.81848100	-4.27112600	-0.00011700
C	-9.65000200	-3.15607800	-0.00015900
C	-9.10206100	-1.88561200	-0.00017700
C	-7.71593400	-1.70242900	-0.00015300
C	-7.19359300	-0.37315600	-0.00017400
C	-6.81917700	0.77010300	-0.00019600
C	-6.46456100	2.16246500	-0.00022900
C	-7.47689900	3.12560800	-0.00034300
C	-7.12185900	4.46285400	-0.00037500
C	-5.77680900	4.79888500	-0.00029200
C	-4.84215800	3.77093900	-0.00018100
N	-5.16549600	2.48329500	-0.00014900
H	-3.77966100	3.99082400	-0.00011400
H	-5.45229500	5.83057300	-0.00031300
H	-7.88483300	5.23111100	-0.00046400
H	-8.51196700	2.81267400	-0.00040500
H	-9.73976600	-1.01144200	-0.00021000
H	-10.7253910	-3.27816000	-0.00017800
H	-9.24236300	-5.26698400	-0.00010300

H	-6.78838000	-4.96891600	-0.00006100
C	-2.08711800	-3.74011000	-0.00001200
C	-0.70660000	-3.71133200	0.00001500
C	-0.04382200	-2.49258800	0.00001800
H	1.03636400	-2.45864000	0.00003900
H	-0.14246500	-4.63417900	0.00003300
H	-2.62165800	-4.68093900	-0.00001500
Br	-3.18861200	0.25250900	-0.00006200
F	-0.26985500	0.74271300	-1.07756400
F	1.32408300	-0.20935400	0.00002700
F	-0.26989400	0.74273200	1.07753500

9-H

G = -3773.008810 hartrees

C	0.00000000	0.00000000	0.00000000
C	1.33454500	-0.68504000	-0.03168000
C	1.35878200	-2.07307600	-0.03922300
C	2.57595900	-2.72673800	-0.03453600
C	3.77950800	-2.01112900	-0.02239000
C	5.05284900	-2.64934000	-0.01818300
C	6.16352400	-3.10898600	-0.01355700
C	7.50056300	-3.60123000	-0.00711200
C	7.75723100	-4.97329100	-0.00085400
C	9.05939000	-5.44363400	0.00597500
C	10.1248690	-4.54933100	0.00650700
C	9.88720200	-3.18523000	0.00019200
C	8.58064500	-2.69365000	-0.00657200
C	8.32983700	-1.28863000	-0.01288600
C	8.07604900	-0.11310600	-0.01832800
C	7.78652200	1.29221200	-0.02542000
C	8.83080700	2.21963900	-0.03737700
C	8.52184300	3.56826000	-0.04454700
C	7.18890500	3.94874600	-0.03951800
C	6.22063900	2.95260100	-0.02735700
N	6.49569400	1.65317300	-0.02040400
H	5.16770500	3.21483600	-0.02311800
H	6.89867600	4.99057000	-0.04483800
H	9.31013200	4.31036800	-0.05404300
H	9.85433200	1.87083200	-0.04097300
H	10.7097390	-2.48218800	0.00056900
H	11.1427000	-4.91721500	0.01186400
H	9.24532800	-6.50981900	0.01096700
H	6.92008800	-5.65905200	-0.00128500
C	3.72790500	-0.61211200	-0.01588400

C	2.51171000	0.04799000	-0.01942100
H	2.47735100	1.12878300	-0.01693500
H	4.65858300	-0.05540300	-0.00954700
Br	2.59365300	-4.61714600	-0.04831600
H	0.43647800	-2.63865500	-0.05360500
F	0.09655900	1.30496000	-0.27247700
F	-0.85223600	-0.53263500	-0.88513000
F	-0.58166500	-0.10840900	1.20355900

9-Br

G = -3773.006924 hartrees

C	0.00000000	0.00000000	0.00000000
C	1.38372000	0.58045900	-0.00003100
C	2.45831400	-0.29902700	-0.00003400
C	3.74857800	0.19704600	-0.00006700
C	3.97890200	1.58163900	-0.00009800
C	5.30253900	2.11229900	-0.00012100
C	6.41998100	2.55762300	-0.00012100
C	7.72938400	3.12307800	-0.00012800
C	7.87603500	4.51346800	-0.00030200
C	9.13186100	5.09376400	-0.00031400
C	10.2678370	4.29113300	-0.00014700
C	10.1405650	2.91350300	0.00003000
C	8.87952700	2.30936700	0.00004000
C	8.79958000	0.88316700	0.00026600
C	8.81229100	-0.31984600	0.00042400
C	8.93777700	-1.75134900	0.00004100
C	10.2139760	-2.32136600	-0.00018300
C	10.3268050	-3.70020500	-0.00050400
C	9.17128500	-4.46631700	-0.00058600
C	7.94731500	-3.80930300	-0.00033400
N	7.82046300	-2.48764600	-0.00002700
H	7.02007400	-4.37273200	-0.00038200
H	9.20979800	-5.54717200	-0.00083400
H	11.3027590	-4.16907700	-0.00068500
H	11.0848060	-1.68030200	-0.00009900
H	11.0177800	2.28001600	0.00017000
H	11.2525180	4.74032100	-0.00015100
H	9.22644500	6.17191400	-0.00045200
H	6.98548600	5.12803100	-0.00043000
C	2.87910100	2.44465300	-0.00010000
C	1.58571500	1.95185400	-0.00006600
H	0.74093700	2.62638200	-0.00006300
H	3.05791000	3.51151800	-0.00012400

Br 5.21409900 -0.98882100 -0.00009600
H 2.29107000 -1.36848300 -0.00001000
F -0.94531500 0.94435500 0.00006700
F -0.21041100 -0.77388000 -1.07392800
F -0.21033200 -0.77395700 1.07388600

Figure S5. NMR Spectra

^{1}H, 400 MHz, $CDCl_3$

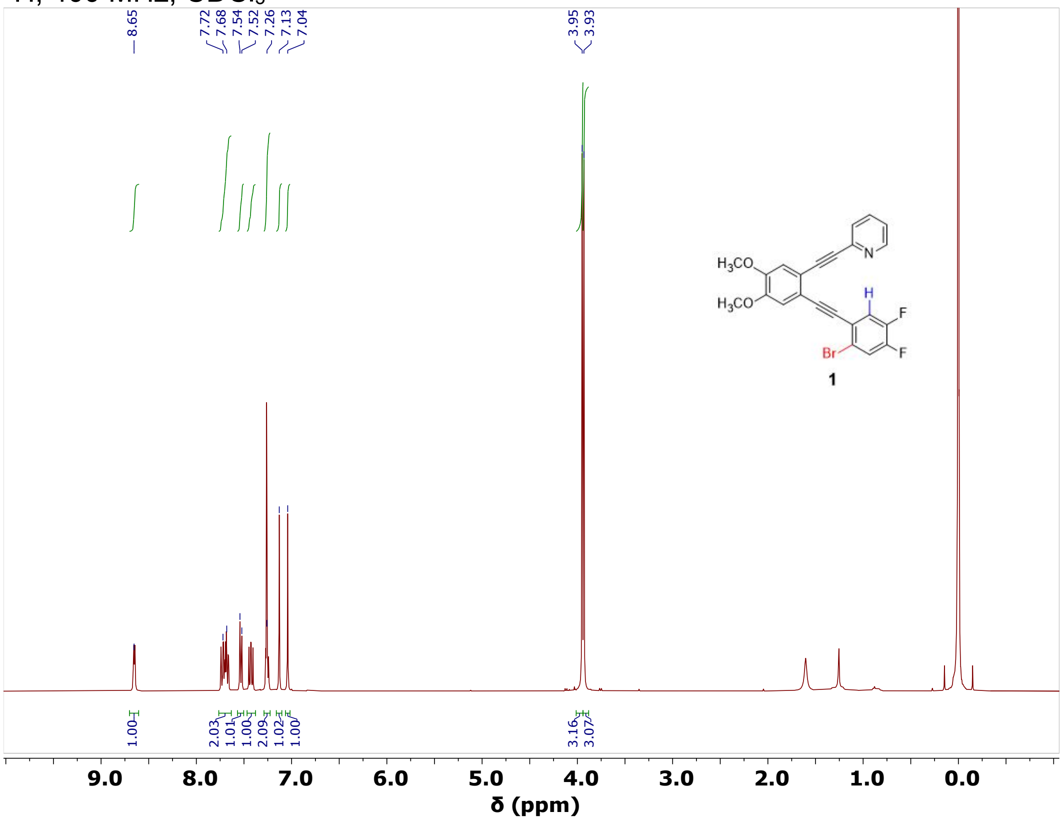

$^{13}C\{^{1}H\}$, 100 MHz, $CDCl_3$ (^{19}F coupling observed)

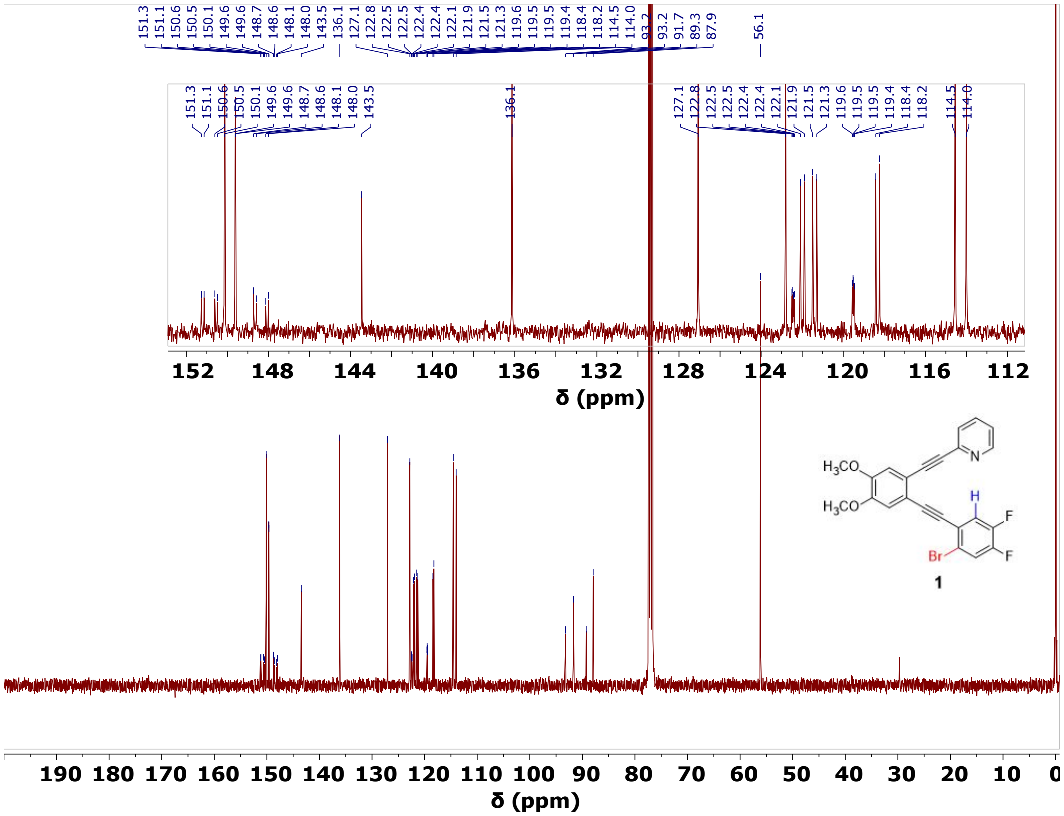

^{19}F, 376 MHz, $CDCl_3$

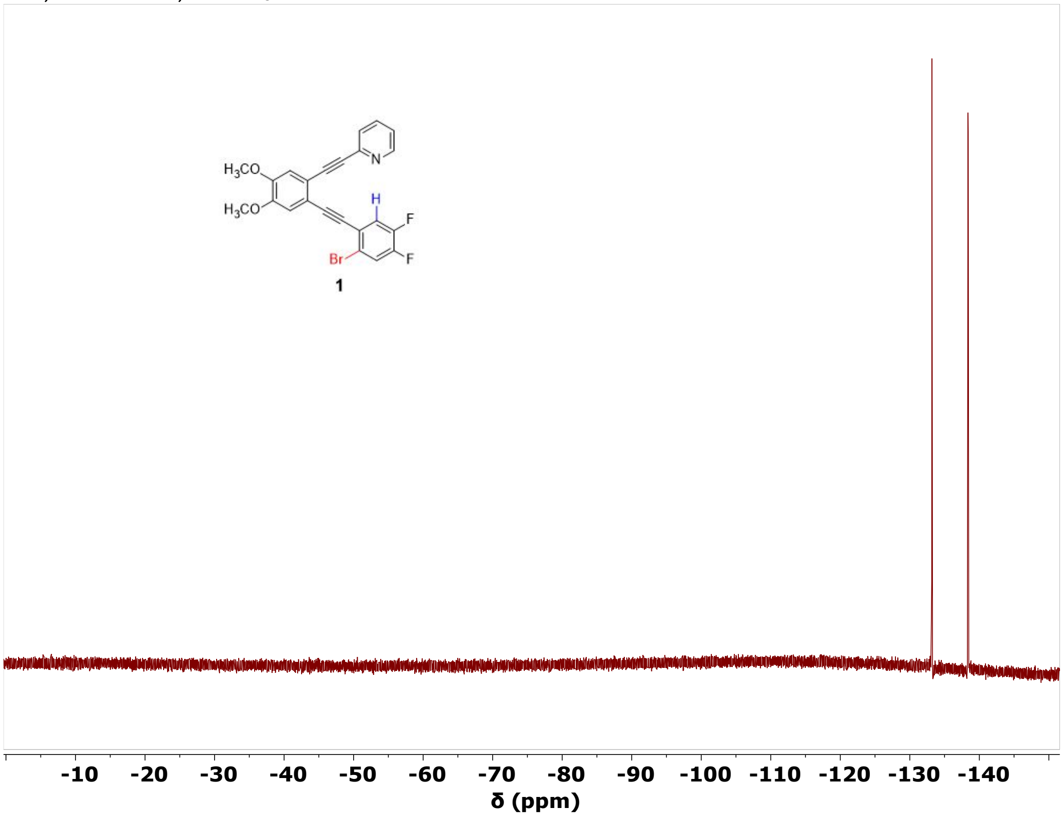

^{1}H, 400 MHz, $CDCl_3$

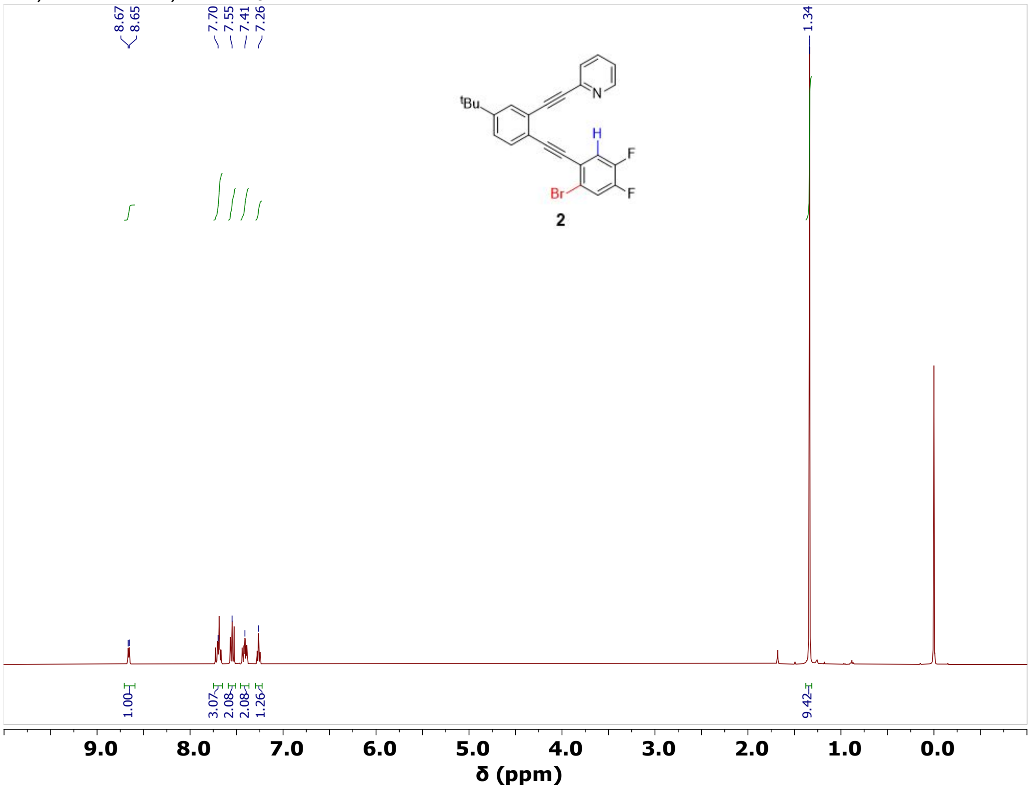

$^{13}C\{^{1}H\}$, 100 MHz, $CDCl_3$ (^{19}F coupling observed)

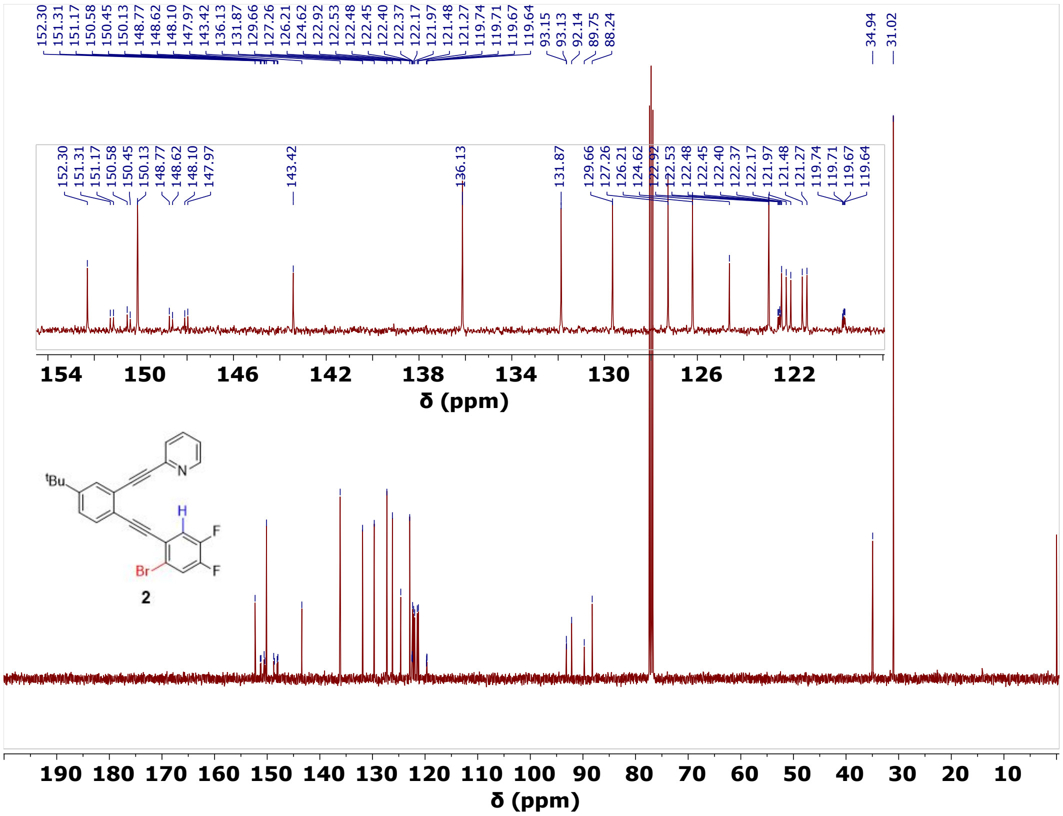

^{19}F, 376 MHz, $CDCl_3$

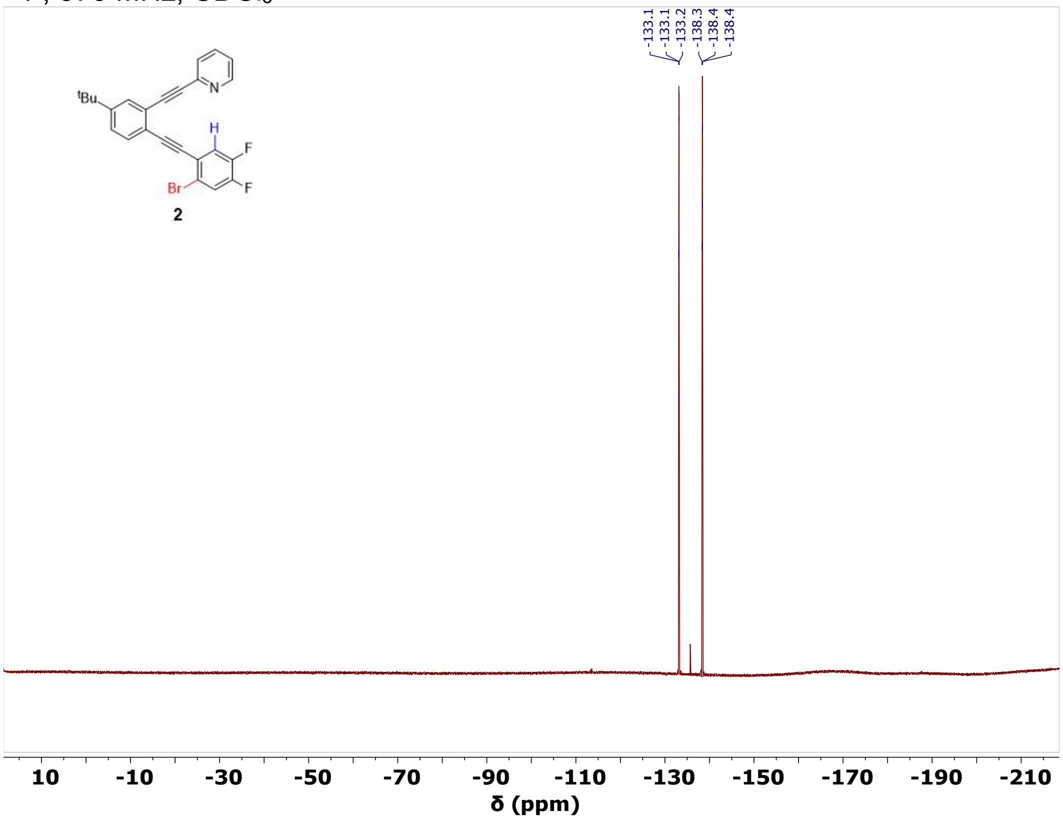

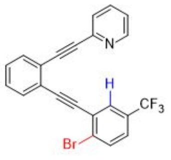

3

^{1}H, 400 MHz, $CDCl_3$

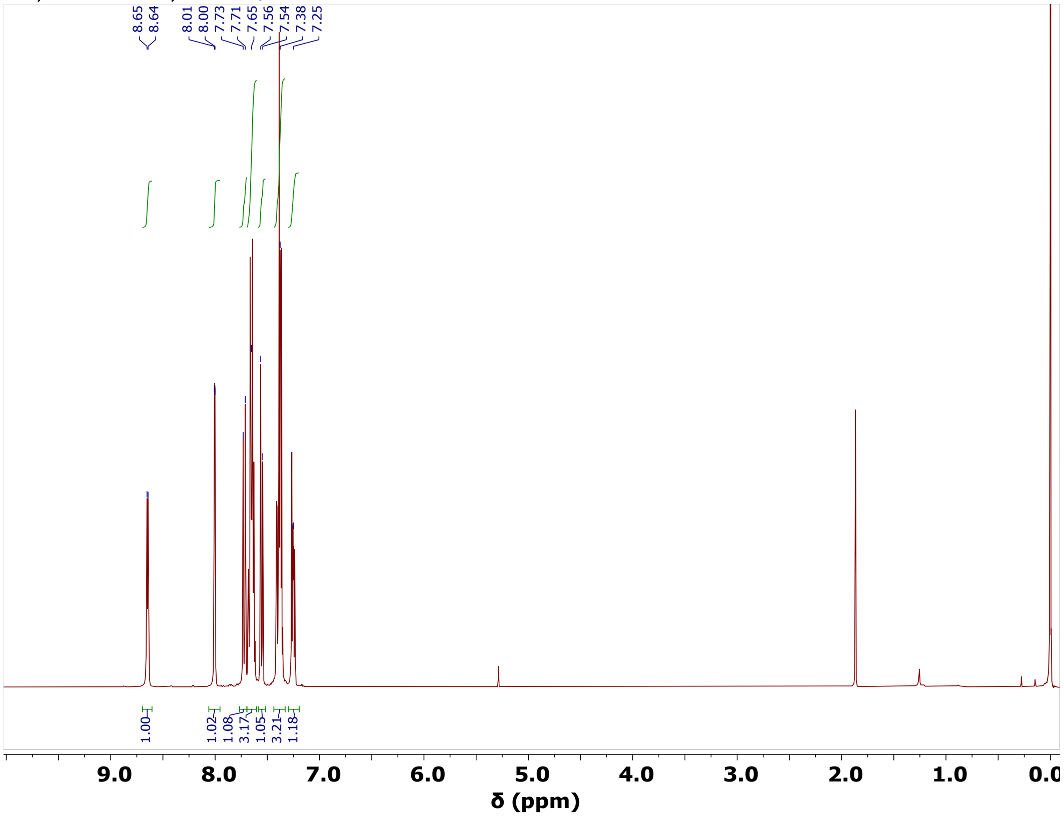

$^{13}C\{^{1}H\}$, 100 MHz, $CDCl_3$ (^{19}F coupling observed)

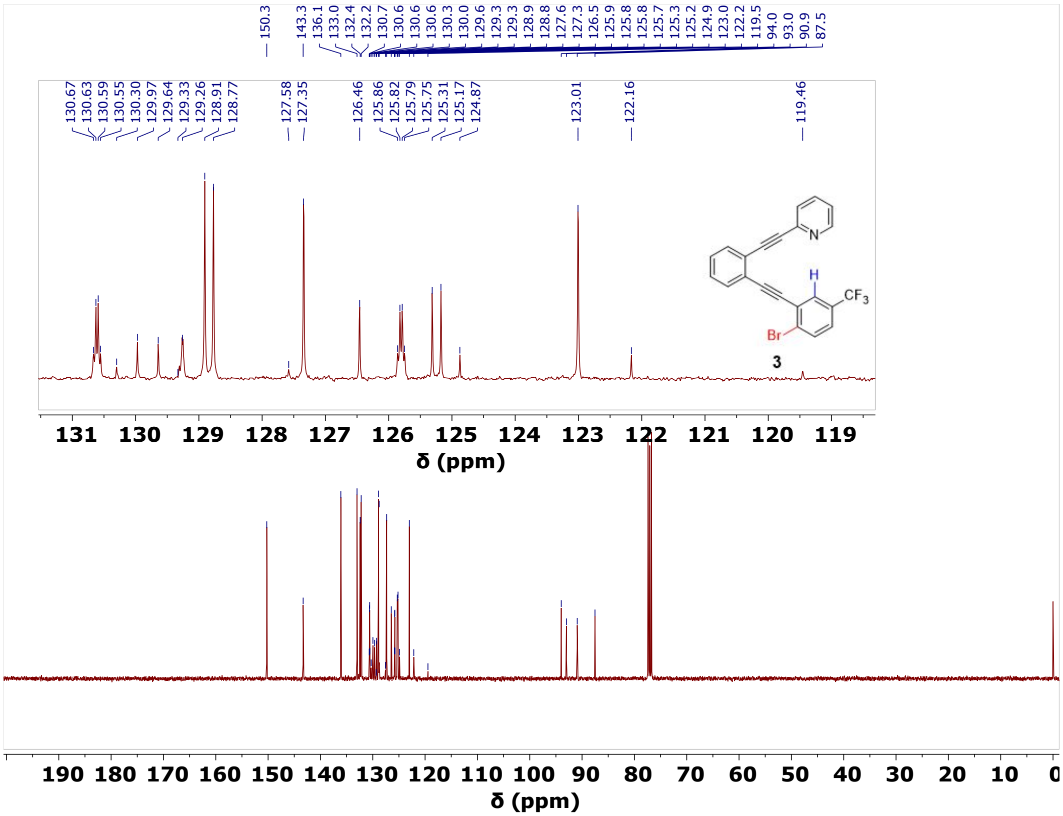

^{19}F, 376 MHz, $CDCl_3$

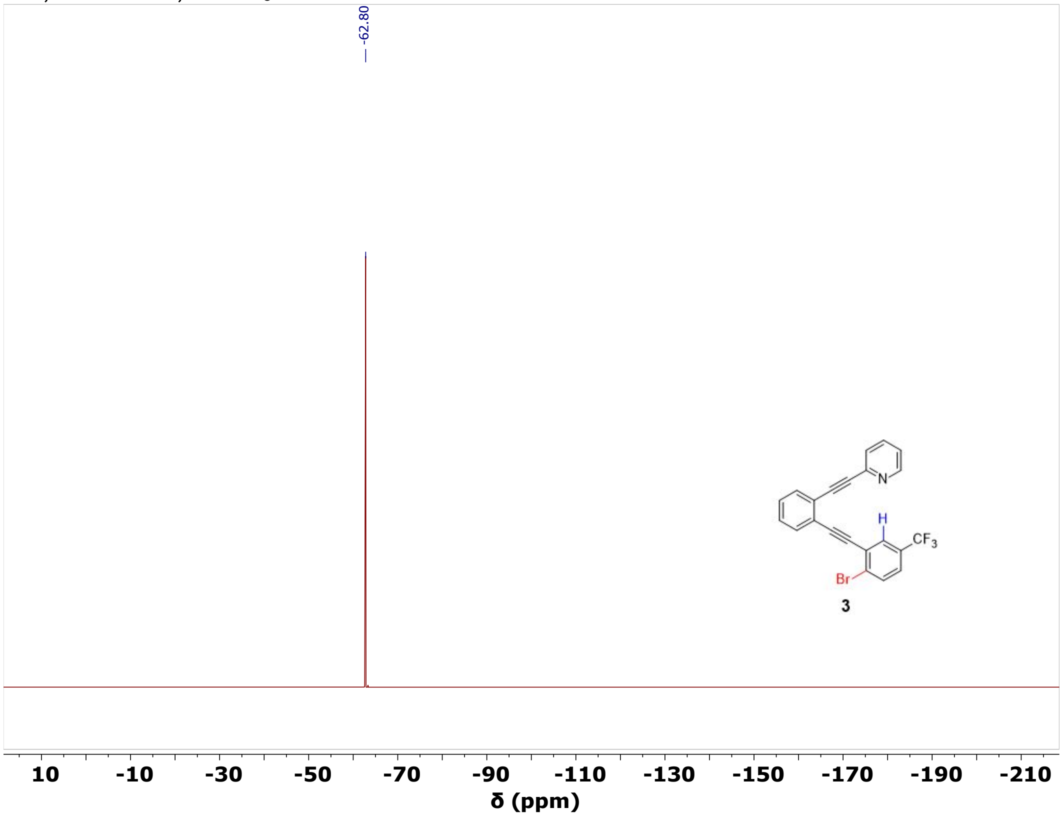

^{1}H, 400 MHz, $CDCl_3$

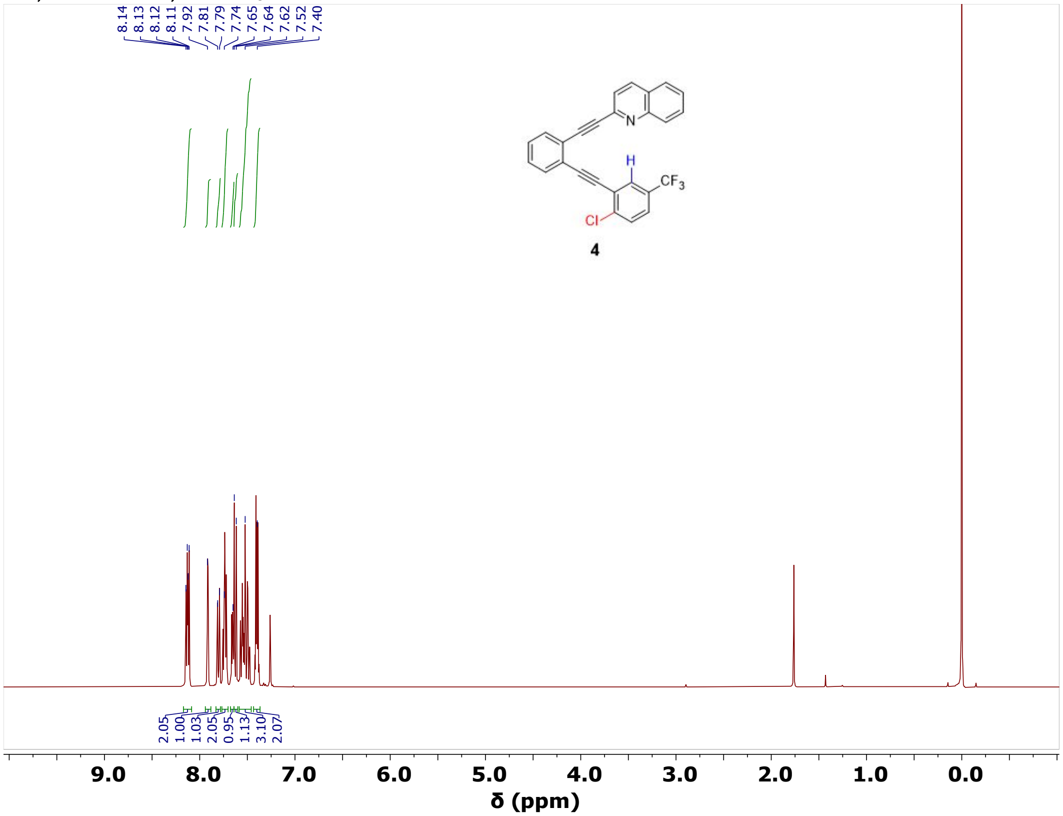

$^{13}C\{^{1}H\}$, 100 MHz, $CDCl_3$ (^{19}F coupling observed)

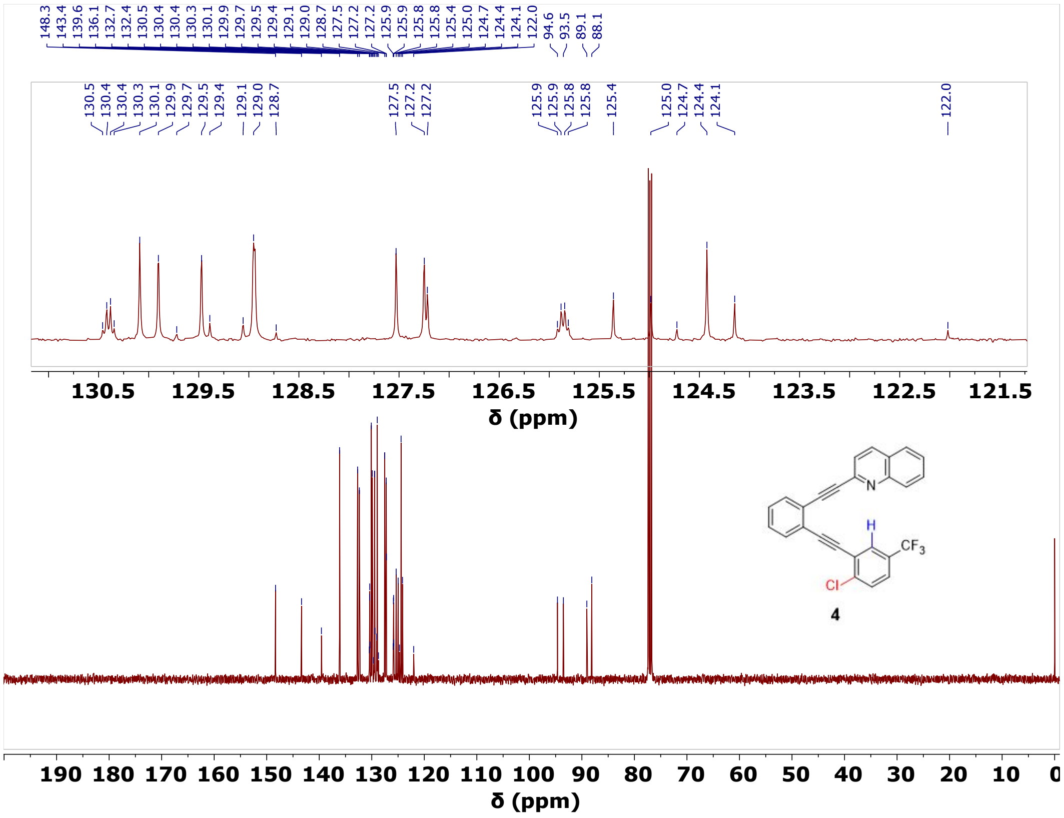

^{19}F, 376 MHz, $CDCl_3$

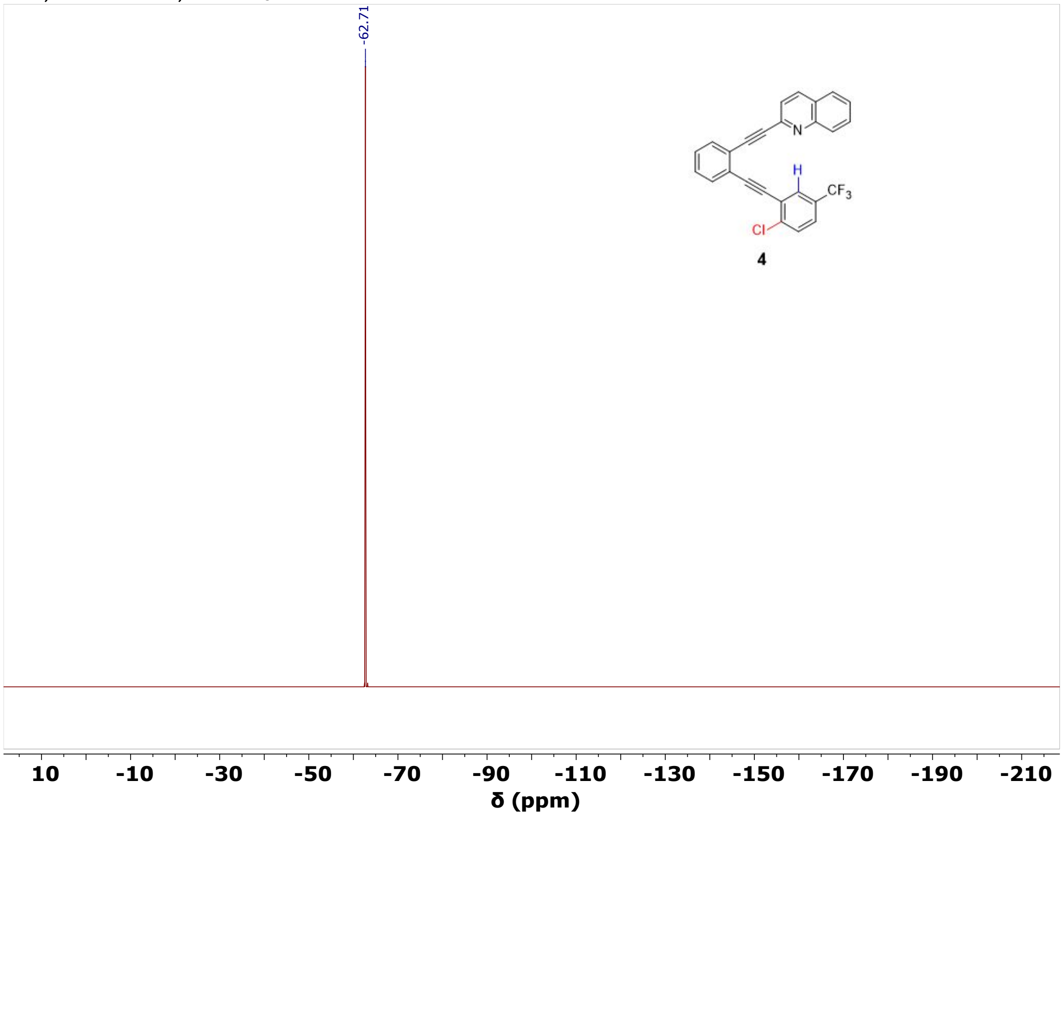

Supplement: Supplementary file 1 [file gg6c00009_si_001.pdf]
